# PAT (Periderm Assessment Toolkit): A Quantitative and Large-Scale Screening Method for Periderm Measurements

**DOI:** 10.34133/plantphenomics.0156

**Published:** 2024-03-29

**Authors:** Gonzalo Villarino, Signe Dahlberg-Wright, Ling Zhang, Marianne Schaedel, Lin Wang, Karyssa Miller, Jack Bartlett, Albert Martin Dang Vu, Wolfgang Busch

**Affiliations:** Plant Molecular and Cellular Biology Laboratory, Salk Institute for Biological Studies, La Jolla, CA, USA.

## Abstract

The periderm is a vital protective tissue found in the roots, stems, and woody elements of diverse plant species. It plays an important function in these plants by assuming the role of the epidermis as the outermost layer. Despite its critical role for protecting plants from environmental stresses and pathogens, research on root periderm development has been limited due to its late formation during root development, its presence only in mature root regions, and its impermeability. One of the most straightforward measurements for comparing periderm formation between different genotypes and treatments is periderm (phellem) length. We have developed PAT (Periderm Assessment Toolkit), a high-throughput user-friendly pipeline that integrates an efficient staining protocol, automated imaging, and a deep-learning-based image analysis approach to accurately detect and measure periderm length in the roots of *Arabidopsis thaliana*. The reliability and reproducibility of our method was evaluated using a diverse set of 20 Arabidopsis natural accessions. Our automated measurements exhibited a strong correlation with human-expert-generated measurements, achieving a 94% efficiency in periderm length quantification. This robust PAT pipeline streamlines large-scale periderm measurements, thereby being able to facilitate comprehensive genetic studies and screens. Although PAT proves highly effective with automated digital microscopes in Arabidopsis roots, its application may pose challenges with nonautomated microscopy. Although the workflow and principles could be adapted for other plant species, additional optimization would be necessary. While we show that periderm length can be used to distinguish a mutant impaired in periderm development from wild type, we also find it is a plastic trait. Therefore, care must be taken to include sufficient repeats and controls, to minimize variation, and to ensure comparability of periderm length measurements between different genotypes and growth conditions.

## Introduction

During plant secondary growth, the periderm arises as a protective layer in roots replacing the epidermis as an outer layer [1]. The periderm is derived from the pericycle, an inner root tissue protected by the cortex, endodermis, and epidermis [1]. The periderm is present in most eudicot plants and is composed of 3 different cell types—phellogen, phelloderm, and phellem [1]. The transit-amplifying cells forming the meristematic-phellogen tissue differentiate bidirectionally: inwardly into the phelloderm and outwardly into the phellem [2]. In species with periderm, phellogen expansion is an important component of plant growth and development [3]. As plants increase in size, the cork cambium (phellogen) promotes growth and safeguards the plant’s inner tissues [2,4].

Extracellular polymers, including suberin, are deposited around the phellem cells, which act as a barrier between the plant and the environment to counter biotic and abiotic stresses [5,6]. The chemical composition (e.g., suberization) of the phellem is associated with the plant’s ability to withstand pathogen penetration and protect against abiotic stress [7]. Suberin is a major secondary cell wall polymer in plants mostly composed of alcohols, hydroxy fatty acids, dicarboxylic acids, ester-linked glycerol, very long-chain fatty acids, and ferulic acid [8]. In the periderm, suberin is deposited between the plasma membrane and the cell wall of the phellem cells forming a lamellar structure displaying alternating light and dark bands [9]. This unique arrangement can be observed using a transmission electron microscope [10]. The orchestration and establishment of root suberin is thought to be mainly controlled by MYB transcription factors [10–13].

Periderm develops over a series of stages in roots. This has been characterized in the greatest detail in *Arabidopsis thaliana*. Prior to periderm formation (stage 0), the pericycle tissue is fully surrounded by 8 endodermal cells and does not divide. In stage 1 of periderm formation, the pericycle starts to divide anticlinally at the xylem poles and extends throughout the pericycle [14]. In stage 2, the first periclinal division takes place in the pericycle forming a partial double periderm/pericycle layer, which is referred to as the phellogen, a secondary meristem. One to 2 endodermal cells are lost at the phloem poles at this stage as they undergo programmed cell death. In stages 3 and 4, which are difficult to distinguish in roots, the pericycle continues to proliferate periclinally. The first phellem cells differentiate from the phellogen in stages 3 and 4. In stage 5, cortex and epidermis tissues break off and are replaced by a mature suberized periderm tissue (stage 6). Although the developmental stages in hypocotyl and root periderm are similar, there are a few differences in the stages; for instance, epidermis and cortex are shed off earlier in roots than in hypocotyls. Due to its late formation during root development, its exclusive presence in the matured regions of the root system and its impermeability, it is one of the most understudied root tissues [14,15].

Much of what is known about periderm development in *A. thaliana* has been learned through root cross-sections [2]. This is a process in which roots are stained and imaged using microscopy at various time points in growth, revealing the anatomy of the periderm as well as highlighting its 3 cell layers comprised of the phellogen, phelloderm, and phellem [1,16]. Another approach is to use Fluorol Yellow (FY) staining on roots to stain suberin present in the cell walls [17,18]. The FY-staining process is a relatively simple and quick procedure and has an advantage in that the dye can be excited with a standard confocal microscope laser. Further, it reveals differences in the cell shape of 2 suberized tissues: the endoderm, with long and rectangular cells, and the periderm/phellem, with smaller and more irregularly shaped and sized cells [7,19].

Another way to measure processes related to periderm formation was employed by using a trait called the phellem length. This trait describes the length of the root that is covered by phellem. The rationale behind this trait is that if periderm formation is delayed or defective, at a given time point, the length of the phellem will be shorter, and if it is accelerated or happens prematurely, the phellem will be longer. Consequently, phellem measurement serves as an indicator of variations in periderm formation when comparing different lines or treatments. This metric has demonstrated its efficacy in quantifying the impacts of mutations on periderm development [1,14].

However, the measurement of phellem length has traditionally relied on human observers, employing a labor-intensive and time-consuming approach. Due to the lack of high-throughput methods to measure periderm, no genetic screens have been conducted to identify genes responsible for periderm formation. However, recent advancements in deep learning techniques, particularly convolutional neural networks, have demonstrated remarkable success in image-based phenotyping [12]. UNet/UNet++, an encoder–decoder-based architecture, has become a popular deep learning model for semantic segmentation of digital images. To address the challenges associated with histological studies, we developed a Periderm Assessment Toolkit (PAT) pipeline to facilitate the high-throughput measurements of periderm length (equivalent to phellem length) in *A. thaliana* roots. For this, we used a streamlined staining protocol to stain roots in an efficient and scalable manner, automated microscopy to facilitate the acquisition of images to capture roots at cellular resolution, and the UNet++ [20] model to automatically detect periderm cells and quantify the length of the periderm in roots. We tested this pipeline on 20 diverse Arabidopsis accessions and a mutant impaired in periderm development and benchmarked its performance. Overall, PAT constitutes a method by which periderm measurements can be performed at a scale suitable for forward genetic screen, such as genome-wide association mapping or screenings of mutant collections.

## Materials and Methods

### Plant materials and growth conditions

We randomly chose 20 *Arabidopsis thaliana* accessions (Table 1). Seeds from these accessions were gas sterilized with 200 ml of commercial bleach and 2.5 ml of hydrochloric acid in a container in a fume hood. Seeds were sown on plates containing 1% agar, 1/2 Murashige and Skoog (MS) medium, 1% sucrose, and pH adjusted to 5.7. The plates with seeds were stratified at 4 °C for 4 d and then moved to a growth chamber (21 °C, 60% humidity, 100 μmol/m^2^/s) and grown vertically.

**Table 1.** List of accessions used in this study

This work ID	Accession ID	Country
1_7	Acc-ID:1829/Mdn-1	USA
2_12	Acc-ID:5921/DraIV 3-7	Czech Republic
3_23	Acc-ID:7424/Jl-3	Czech Republic
4_18	Acc-ID:9558/IP-Moc-11	Spain
5_24	Acc-ID:9904/IP-Vas-0	Spain
7_18	Acc-ID:9892/IP-Sam-0	Spain
13_3	Acc-ID: 5748/Kil-0	UK
15_27	Acc-ID: 6149/T970	Sweden
16_34	Acc-ID: 6276/TV-30	Sweden
17_23	Acc-ID: 6909/Col-0	USA
18_8	Acc-ID: 6956/Pu2-7	Czech Republic
19_3	Acc-ID: 7014/Ba-1	UK
20_29	Acc-ID: 2106/MSGA-10	US
21_12	Acc-ID: 7333/Sei-0	Italy
22_22	Acc-ID: 7223/Li-2:1	GER
23_12	Acc-ID: 7515/RRS-10	US
25_8	Acc-ID: 8427/Ull2-13	Sweden
26_15	Acc-ID: 9382/Fri 2	Sweden
27_10	Acc-ID: 9452/Spro 3	Sweden
28_11	Acc-ID: 9532/IP-Cdo-0	Spain

### Experimental and technical design

#### Root staining and image generation

**Fig. 1.**
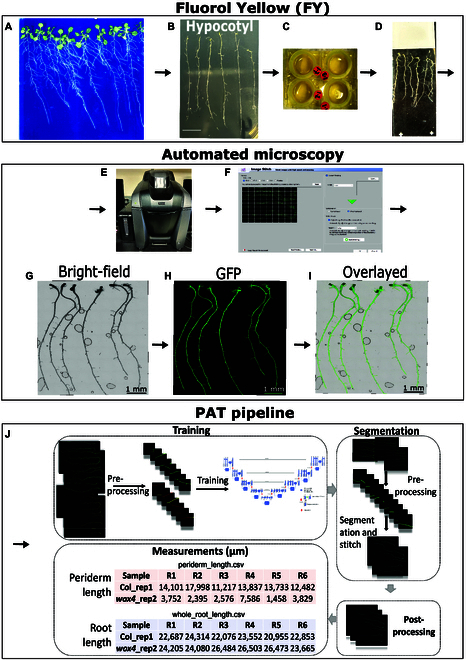
Overview of root processing and PAT pipeline. (A) Seedlings are grown vertically for 14 d on 1/2 MS plates. (B) Lateral roots are removed, and the upper part of the root (~3 cm down from the hypocotyl) is kept. (C) Roots are stained with FY. (D) Six roots are mounted onto a single slide. (E) The FY-stained roots are then scanned by an automated digital microscope (Keyence). (F) Images generated are manually stitched. (G to I) TIFF images are generated in bright-field (left) and green fluorescent protein (GFP) channels (middle), and overlayed (right). (J) Images are automatically processed and analyzed: firstly, during training phase, images were annotated to generate the ground truth for training using the Image Polygonal Annotation with Python program (https://github.com/wkentaro/labelme) and then the original images and annotated images were used as inputs to train a UNet++ model with the ResNet encoder. For automated segmentation, periderm, endoderm, and lateral roots masks were generated using the trained model. Postprocessing of the images involved the elimination of inaccurately segmented periderm gaps using a threshold of 150 pixels. A Python script was utilized to measure the length of both periderm and root from the hypocotyl downwards, outputting measurements in both micrometers and pixels. The variable “R” denotes the number of roots.

Seedlings were harvested at 14 d after the plates had been placed in the growth chamber (Fig. 1A). As the periderm is in the old part of the root, the younger parts of the root, as well as lateral roots, were removed while the upper part of the primary root, ~3 cm down from the hypocotyl, was collected (Fig. 1B). These root portions from the 20 accessions were stained with freshly prepared Fluorol Yellow 088 (Fisher Scientific and Santa Cruz Biotechnology Fluorol Yellow 088, 81-37-8) (0.01% w/v, in lactic acid, Sigma-Aldrich) at 70 °C for 30 min and subsequently rinsed in water 3 times for 5 min [17,18] (Fig. 1C)*.* Six roots per accession were then mounted onto a single microscopy slide (Fisher Scientific, size 25 × 75 × 1.0 mm) with a cover slip (VWR micro cover glass, size 22 × 40 mm) (Fig. 1D). The microscopy slides were then transferred facing down to a Keyence microscope, BZ-X810 All-in-One Fluorescence Microscope (https://www.keyence.com/products/microscope/fluorescence-microscope/bz-x700/models/bz-x810/) (Fig.1E). Using the capture still images (sample holder “Slide”) function of the Keyence software, roots were first scanned with the 2× objective using the “Navigation” tool. Images are autofocused for clarity, points are set for field of view, and images are subsequently scanned and acquired using a 4× objective with the bright-field and green fluorescent protein channels (BZ Series infinite optical system filters). The images acquired were then manually stitched using the stitching function “image stitching of multiple fields of view” uncompressed (Fig.1F). Stitched images were then saved as tag image file format (TIFF) images (Fig. 1G to I). These TIFF images were then used for further image processing (Fig. 1J).

#### Evaluating the precision of the periderm detection pipeline

**Fig. 2.**
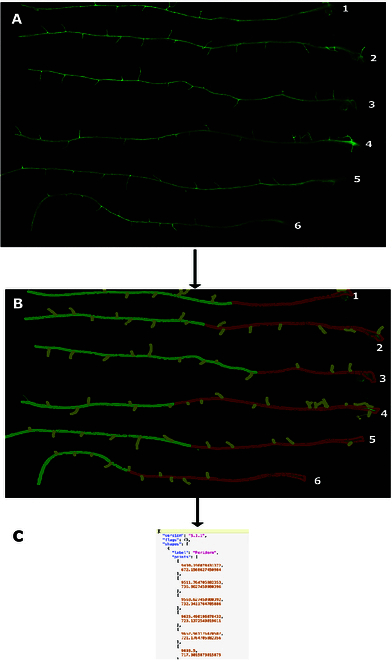
Manual versus pipeline segmentation benchmark. (A) Upper panel shows an image of 6 roots stained with FY that was acquired by automated microscopy. The hypocotyl is indicated by the number next to each root. (B) Same roots manually labelled containing 3 classes of pixels for tissue type: periderm (red), endoderm (green), and lateral roots (yellow). (C) These labeled images are saved as JavaScript object notation files, which are then used to quantify the accuracy of PAT in periderm detection.

To test PAT’s accuracy in detecting periderm over other root tissues (endoderm and lateral roots), we benchmarked pixel-level segmentation accuracy with ground-truth manual annotation using precision, recall, and F1 score. We first manually annotated ground truth labeling for tissue type (periderm, endoderm, and lateral roots) on Portable Network Graphic (PNG) images. We used 3 microscopy slides, each one containing 6 roots previously stained with FY. The microscopy slides we used for this benchmarking contained roots from the line SALK_025743. Using the “create polygon” function, we labeled the boundary of each separate root type in the program “Image Polygonal Annotation with Python” (https://github.com/wkentaro/labelme) by manually tracing the boundary of periderm, endoderm, and lateral roots using a segmented lines. These manually segmented PNG images were saved into the JavaScript object notation files and used to benchmark the accuracy of the pipeline. Figure 2 shows one microscopy slide with 6 roots, stained with FY, and labeled with our annotation.

#### Image annotation and preprocessing

The segmentation model and analysis pipeline were developed and trained on the high-performance computing platform, run:ai (https://www.run.ai/), using 2 graphics processing units (2X NVIDIA A40 graphics processing units [GPUs]) with 80 GB of memory. To train the pipeline, we used 9 microscopy TIFF images containing roots from Col-0 and the line SALK_025743 (54 roots total). The microscopy TIFF images underwent several preprocessing steps. Firstly, the images were converted from TIFF to PNG format using the “ffmpeg” library (https://github.com/wkentaro/labelme), which facilitated using TensorFlow and PyTorch frameworks. The converted PNG image files were then used for manually annotated ground-truth labeling based on tissue type (periderm, endoderm, and lateral roots), and other pixels were defined as background. Since the FY microscopy images are large (~ 14,000 × 10,800 pixels), thereby exceeding the available GPU memory for subsequent training, the images were cropped into ~1,471 smaller images each with a size of 1,024 × 1,024 × 3 pixels (where 3 refers to red, green, and blue channels). Data augmentation (flipping and rotation) was applied to training patches to compensate for deficient background and blurry images.

#### Deep learning model training

We employed the UNet++ architecture with ResNet encoder for image segmentation, which is identification of tissue type in this study. The UNet++ model was implemented with 1,471 small patches of original images and annotation images, while 60% of the patches (i.e., 883 small patches) were used to train the model, 20% were used to validate the model, and the remaining 20% were used to test model performance. The model was optimized using Adam optimizer with an initial learning rate of 0.0001 and epochs of 40. The loss function of this model summed up with the logits loss and dice loss, and performance evaluation metrics was interaction over union score. The best validated model with the highest interaction over union score was saved as a well-trained model.

#### Predictions

During the prediction phase, 20 images of different accessions were randomly selected. As the original images are too big to process, we did the same preprocessing as in the training phase, which is to crop the images into small patches that are used for periderm segmentation based on the well-trained UNet++ framework. To segment small patches, the inputs of the well-trained UNet++ model were small patch images, and the outputs of the model were segmented images, which indicates the tissue type of each pixel in patch images.

#### Image postprocessing, periderm measurement, and PAT pipeline usage

**Fig. 3.**
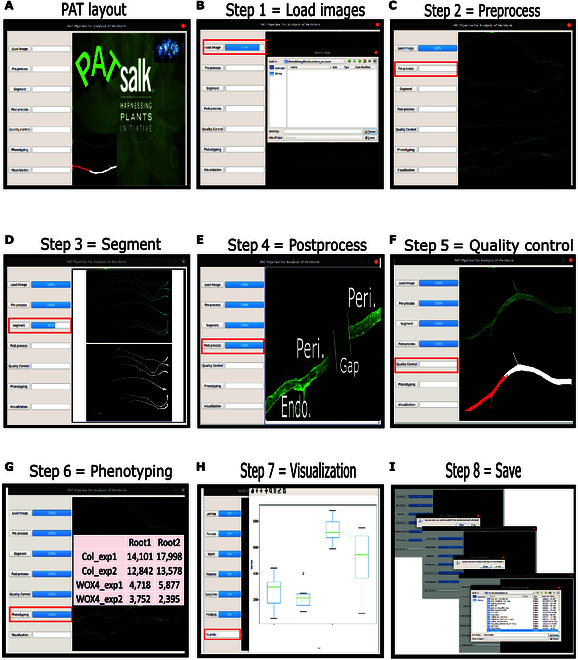
Layout of the PAT pipeline run in Linux. (A) PAT interface. The PAT pipeline features an intuitive user interface with a set of clearly labeled buttons for executing each step of the analysis. Progress bars (depicted by blue squares) provide visual feedback on the ongoing processes, and a dedicated window displays command execution for transparency. (B) Step 1. Load images: In this initial step, users can conveniently select the folder containing images for analysis. The loaded images are displayed in a right-hand panel, and a progress bar indicates the loading progress. (C) Step 2. Preprocess: Image format compatibility is crucial for analysis. If a dataset includes non-PNG images (e.g., TIFF, PNG, and JPEG), the preprocess option allows for on-the-fly conversion to the PNG format, ensuring uniformity in the analysis. (D) Step 3. Segment: This step involves segmentation to isolate and identify roots and periderm regions within the images. (E) Step 4. Postprocess: This step rectifies gaps within continuous periderm zones, which may result from microscopy errors (e.g., blurriness). If a gap emerges within a continuous periderm zone (and if the gap is <150 pixels), the postprocessing step connects the periderm regions on either side of the gap, ensuring a smooth integration. If the gap is >150 pixels, the predicted periderm is considered a false positive (Peri, periderm; Endo, endoderm). (F) Step 5. Quality control (QC): The QC step generates vertical images for each root from the original data and the segmented results. This allows users to select high-quality images, and a text file containing the selected images is generated. (G) Step 6. Phenotyping: The periderm phenotyping step is essential for measuring the length of periderm. PAT generates 4 comma-separated values (CSV) files as outputs: “periderm_length_micrometers.csv”,“periderm_length_pixels.csv”,“whole_root_length_micrometers.csv”, and “whole_root_length_pixels.csv”. Moreover, if QC is executed, PAT provides additional output files titled “periderm_length_after_QC_micrometers.csv” and “periderm_length_after_QC_pixels.csv”. (H) Step 7. Visualization: This step generates boxplots for each slide, aiding in the visual representation of the data. Users can save these plots for further analysis and reporting. (I) Step 8. Save: To exit the GUI interface, users can simply press the “Esc” key. Upon exiting, a prompt will inquire whether to save the results and specify the desired save location, ensuring data preservation and convenience. Red squares in panels highlight the steps in the pipeline.

The segmented periderm images are further refined and processed through Python scripts that are integrated within the PAT pipeline (Fig. 3). This postprocessing step enhances the accuracy of periderm measurements by addressing any contextual errors and optimizing the final results. This involved: (a) stitching together the segmented patches of all images in one slide, (b) connecting small periderm prediction segments of each root, (c) closing gaps within the periderm, and (d) measuring the periderm length per root (Fig. 3E and Fig. S1). The segmented smaller images (patches) were stitched into a final segmentation image that was of the same size as the original image. To connect small periderm segments which had gaps of wrongly segmented pixels, small gaps (less than 150 pixels) were connected to form the periderm. However, if a large gap (>150 pixels) was detected, it was considered a false-negative periderm prediction segment and it was relabeled as endodermis. After separating the periderm from other tissue types, the tiny gaps (less than 20 pixels) within the periderm segments were closed (Fig. 3). The continuous periderm segments were converted into contours and the Euclidean distance was calculated based on the center line of a contour and was saved as the periderm length (in both pixels and micrometers) of each root. To access the PAT pipeline and its scripts, please refer to the GitHub repository at:

https://github.com/Salk-Harnessing-Plants-Initiative/PAT-Pipeline-for-Analysis-of-Periderm. This repository contains the comprehensive set of tools and scripts essential for implementing PAT.

#### Manual periderm measurements

**Fig. 4.**
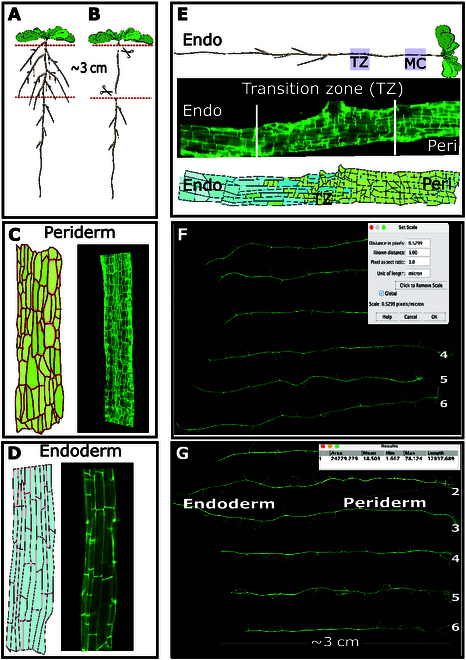
Overview of manual periderm measurements. (A) Arabidopsis accessions are grown for 14 d. (B) The upper portion of the root (~3 cm) is excised alongside with the removal of lateral roots. (C) FY TIFF root images provide a visualization of the periderm, showcasing smaller and irregularly shaped cells (illustrative format and the actual FY image are shown). (D) The FY images also outline the endoderm, characterized by long and rectangular cells (illustrative format and the actual FY image are shown). (E) Similar to the PAT pipeline, we manually measured the MC in the old periderm and the younger periderm toward the root tip, encompassing the periderm within the transition zone (TZ) (illustrative format and the actual FY image are shown). Both MC and TZ are highlighted in the root (purple square) (Peri, periderm; Endo, endoderm). (F) Images are loaded into Fiji and parameters are configured including distance in pixels (0.5299), known distance (1.00), pixel aspect ratio (1.0), and unit of length (micrometers, μm), followed by selecting the Global option. (G) Manual measurements are performed using the “segmented” line tool in Fiji, tracing the periderm from the hypocotyl down to approximately 3 cm from the root's end. The segmented (yellow) line is utilized for measuring the periderm in the roots.

Fourteen-day seedlings are grown (Fig. 4A), and the upper part of the root (~3 cm) is excised with the simultaneous removal of lateral roots (Fig. 4B). Subsequently, roots were stained with FY to highlight 2 distinct suberized cell types: the periderm/phellem exhibiting smaller and irregularly shaped cells (Fig. 4C) and the endoderm characterized by long and rectangular cells (Fig. 4D). Similar to the PAT pipeline, we manually measured the mature cork (MC) in the old periderm and the younger periderm toward the root tip, encompassing the periderm within the transition zone (Fig. 4E). Images of roots stained with FY are captured through automated microscopy and imported into the Fiji image processing software [20]. Utilizing the “Analyze” and subsequently the “Set Scale” function within Fiji, we established a manual scale for measuring periderm dimensions in metric units. This involved inputting specific parameters: (a) pixel distance: 0.5299, (b) known distance: 1.00, (c) pixel aspect ratio: 1.0, (d) length unit: micrometers (μm), followed by selecting the Global option (refer to Fig. 4F). In the FY-stained images, the periderm can be followed along the length of the root from the hypocotyl downward using the segmented line tool in Fiji. Upon encountering a transition from periderm to endodermis, we left the segmented line tool pointer on that spot and pressed “M” to get a measurement of the segmented line. The output, indicating the length of the periderm in micrometers (e.g., 12,837.689 μm) was displayed and saved (Fig. 4G).

#### Segmentation model evaluation metrics

Precision, recall, and F1 score are common metrics used to evaluate the performance of the segmentation models. Precision measures the accuracy of the model in predicting positive instances as shown in Eq. 1. Recall measures the ability of the model to detect positive instances as shown in Eq. 2. F1 score is the harmonic mean of precision and recall, providing a balance between the 2 metrics (Eq. 3).

$$\text{Precision}=\frac{\mathrm{TP}}{\mathrm{TP}+\mathrm{FP}}$$ (1)

$$\text{Recall}=\frac{\mathrm{TP}}{\mathrm{TP}+\mathrm{FN}}$$ (2)

$$F1\;\text{score}=\frac{2\times \text{Prec}i\text{sion}\times \text{Recall}}{\text{Precision}+\text{Recall}}$$ (3)

where TP means true positive, FP means false positive, and FN means false negative.

#### Quality control on the microscopic images

To identify outliers, we incorporated a quality control (QC) step into PAT (Fig. 3F and Fig. S2). For this we used a custom graphical user interface (GUI) tool in which the classifications of different root segments could be evaluated by a user. Given the extensive size of each root image, detailed inspection within a GUI window can be challenging. To mitigate this issue, we isolated each root from its background, allowing us to assess image quality and segmentation results more effectively. Our methodology employed a UNet++ model to segregate roots from the background within binary images. Based on their respective positions in the binary segmented images, roots were then extracted from the original images and images segmented with periderm. Owing to the elongated nature of the roots, displaying all details without zooming in was not feasible. Therefore, we cropped 1,500 pixels on either side of the transition point from periderm to endodermis for each root (Fig. 3F and Fig. S2). Subsequently, we assembled the original and periderm-segmented roots vertically, cropped as described earlier, to generate concatenated images. This process facilitated the inspection of image quality, particularly at the periderm transition position, and segmentation quality within GUI windows. We utilized these merged images as input for our GUI-based image selector, an effective tool for choosing high-quality images with accurately segmented periderm. This is a process that takes a user a few seconds per image and ensures precise measurements in subsequent analyses.

#### PAT reproducibility

To evaluate the consistency and reproducibility of periderm length measurements generated by the PAT pipeline, a selected subset of the 20 accessions listed in Table 1 (1_7, 2_12, 7_18, 15_27, 20_29, and 22_22) underwent a replication process. These 6 accessions were grown for 14 d (conditions specified in Plant materials and growth conditions), stained with FY for visualization, and subsequently analyzed using the PAT pipeline (Figs. 1 and 3 provide a detailed methodology for sterilization and PAT processing of these subset accessions).

#### Genotyping

To gauge replicability, we also run PAT in the *WOX4* (AT1G46480) mutant (SALK_210239). Genotyping for the homozygote *wox4-1* mutant involved the use of the primers K133-WOX4-53B-LP (5′ AGGTCTACCCCCTTTTCAACG), K134-WOX4-53B-RP (5′ AATGTGTGGGTTCAGTTGGAG), and 72-LBb1.3 (5′ ATTTTGCCGATTTCGGAAC). Genomic DNA extraction was conducted as previously described [21]. To assess the reliability and reproducibility of PAT analysis utilizing the *wox4-1* mutant line, 2 independent experiments were conducted and labeled as “Exp.1” and “Exp.2.” Exp.2 was performed 1 week later than Exp.1, introducing a temporal dimension to the experimental design. Supplementary material (Fig. S4) has been included in this manuscript, comprising 4 microscopy images generated from the output of Keyence microscopy. These images correspond to 6 roots each from 2 sets of images in TIFF format: one from the wild-type Arabidopsis (Col-0) for both Exp.1 and Exp.2, and the other from the *wox4-1* mutant for Exp.1 and Exp.2. This collection of images serves as a resource for users to thoroughly evaluate and test the reliability of the PAT pipeline.

## Results

### Periderm segmentation benchmark

**Fig. 5.**
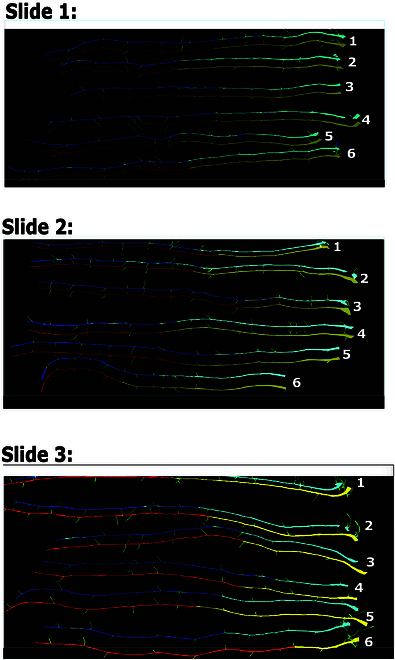
Accuracy of PAT’s periderm segmentation (in pixels) on 18 roots (3 microscopy slides, 6 roots each). Manual segmentation is shown in yellow (periderm), red (endoderm), and green (lateral roots). Automated segmentation is shown in light blue (periderm) and dark blue (endoderm) and green (lateral roots). Root number is indicated in white next to the hypocotyl.

To automate periderm length measurements, we developed a PAT, a pipeline that automatically measures the periderm in Arabidopsis roots (Fig. 3). First, to evaluate PAT’s ability to accurately detect periderm among different root tissue types (periderm, endoderm, and lateral roots), we conducted a benchmarking study. Specifically, we compared the periderm segmentation results generated by the PAT pipeline with expert labeled ground truth data for the corresponding root tissue type, measured in pixels. We used 18 FY-stained roots for this comparison (see Materials and Methods) and benchmarked the pixel-level classification using precision, recall and F1 score to evaluate the efficacy and sensitivity of periderm detection. Our model achieved a precision of 90%, a recall of 86%, and F1 score of 88% in periderm detection after postprocessing (Table 2 and Fig. 5).

**Table 2.** Average scores of the precision, recall, and F1 scores of the segmentation model in detecting periderm

Slide 1	Precision	Recall	F1 score
Before postprocessing	0.854	0.869	0.861
After postprocessing	0.904	0.865	0.884
			
Slide 2	Precision	Recall	F1 score
Before postprocessing	0.863	0.861	0.862
After postprocessing	0.885	0.820	0.851
			
Slide 3	Precision	Recall	F1 score
Before postprocessing	0.875	0.861	0.868
After postprocessing	0.917	0.823	0.867

### Periderm length benchmark in 20 accessions

**Fig. 6.**
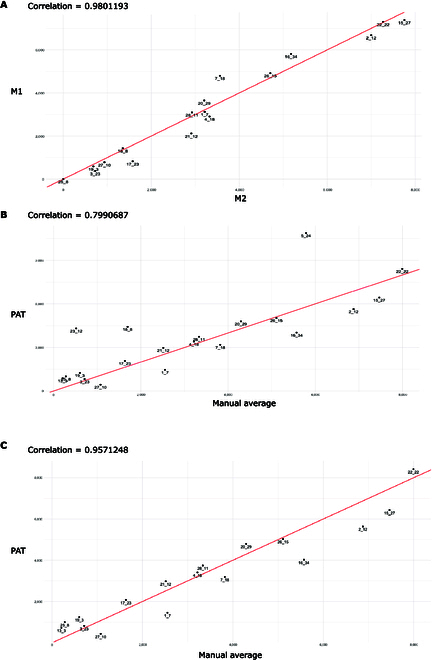
Correlation pattern within periderm length measurements. (A) Scatter plots illustrating the correlation between periderm length measurements from 2 human experts (represented as M1 and M2). (B) Correlation analysis comparing the average measurements of the 2 individuals with PAT before QC. (C) Correlation analysis between the average measurements of the 2 individuals and PAT after QC.

After demonstrating good accuracy of the PAT in differentiating periderm from other root tissues (Table 2), we proceeded to utilize the same pipeline to measure the length of the periderm in 20 diverse natural accessions (Table 1). Our objective was to compare periderm length measurements (in micrometers) with those obtained by human experts as a benchmark, thereby evaluating the effectiveness of the pipeline in measuring periderm length in a set of diverse accessions. For this, we removed lateral roots and kept the upper ~3 cm of the taproot from the hypocotyl down. We then obtained phenotypic images using automated digital microscopy with FY root staining (Fig. 1). We first conducted a correlation analysis of the periderm measurements. To establish a baseline for the potentially best outcome, we had 2 independent expert users measure the periderm of the roots. As expected, the Pearson correlation between the measurements was very high (0.98, Fig. 6A). We then compared PAT’s prediction to the average values obtained by the 2 expert users; we found that the correlation was significant but was not very high (0.79, Fig. 6B) and contained several outliers (roots from accessions 5_24, 23_12, and 18_8; Fig. S3). The presence of the outliers prompted us to develop an efficient QC step. This efficient GUI allows a user to quickly zoom in on the transition zone between the periderm and endoderm. If this periderm transition zone is not detected due to the presence of blurry or poorly captured images, the roots in question are excluded from the analysis (Fig. 3F). As a result of the QC process, we successfully removed outliers consisting of blurry images (5_24, 23_12, and 18_8). Overall, this QC step improved the accuracy of PAT significantly as the correlation of expert user measurements and PAT measured periderm lengths increased by 16% (to 0.95; Fig 6C). Tables S1 and S2 display periderm length values for all the accessions before and after the QC process, respectively.

**Fig. 7.**
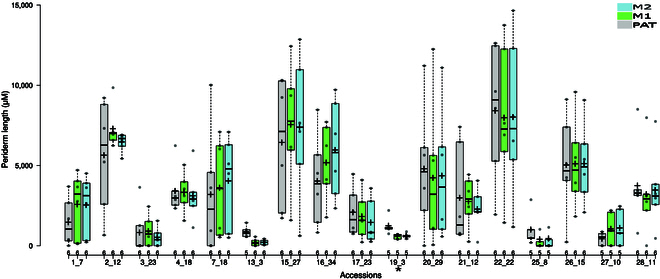
Comparison between PAT and manual measurements of periderm length was conducted on 20 scanned microscopy images, following the implementation of a QC process. The results obtained from PAT are displayed in gray, while manual measurements from 2 human experts (M1 and M2) are shown in green and blue, respectively. QC identified and excluded poorly captured images from accessions 5_24, 18_8, and 23_12, rendering it impossible to conduct periderm length measurement on those slides by pipeline or manual approach. Post-QC, there were no statistical discrepancies observed in 16 out of 17 accessions, resulting in 94% efficiency. The accessions that exhibited differences between the 2 methods are denoted by an asterisk.

We then compared whether the level of PAT’s accuracy can provide an accurate estimate of the periderm length within and between different accessions. For all but one accession, the distribution of periderm lengths of the biological replicates was statistically indistinguishable between the experts’ and PAT’s periderm measurements. This represented 94% efficiency in measuring the periderm length, when considering typical experimental designs for many genetic analyses where multiple individuals of a genotype are measured and compared (Fig. 7 and Table 3). The accession not accurately measured by PAT (accession 19_3), was represented by only 5 roots and showed a minimal amount of periderm.

**Table 3.** Analysis of variance for all the accessions comparing manual versus pipeline measurements after implementing QC

Samples	Effect	DFn	DFd	F	*P*
1_7	Benchmark	2	15	0.749	0.49
13_3	Benchmark	2	15	0.714	0.506
15_27	Benchmark	2	15	0.132	0.877
16_34	Benchmark	2	15	0.758	0.486
17_23	Benchmark	2	15	0.297	0.747
19_3	Benchmark	2	12	5.65	0.019
2_12	Benchmark	2	15	0.881	0.435
20_29	Benchmark	2	15	0.03	0.97
21_12	Benchmark	2	15	0.169	0.846
22_22	Benchmark	2	15	0.017	0.983
25_8	Benchmark	2	12	1.61	0.241
26_15	Benchmark	2	15	0.002	0.998
27_10	Benchmark	2	12	0.747	0.495
28_11	Benchmark	2	15	0.069	0.934
3_23	Benchmark	2	15	0.216	0.808
4_18	Benchmark	2	15	0.07	0.933
7_18	Benchmark	2	15	0.099	0.906

### PAT is able to detect abnormal periderm development

**Fig. 8.**
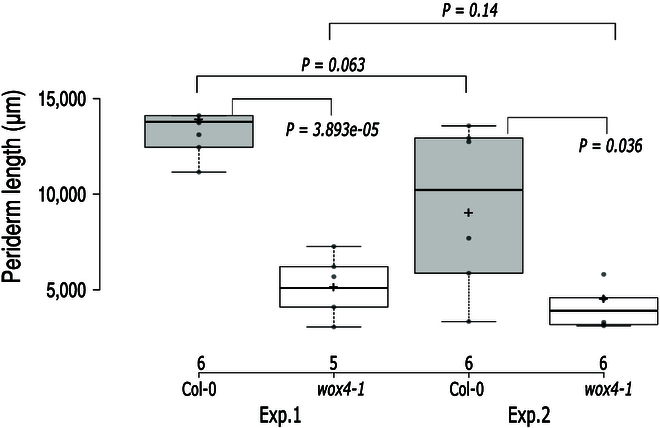
PAT analysis outcomes showcasing a comparative assessment between the mutant line *wox4-1* and the Col-0 wild-type control, conducted across 2 independent experiments labeled as Exp.1 and Exp.2. The significance of the differences observed in each comparison is quantified by the *P* value (*P*) derived from the 2-sided *t* test, reported alongside their respective comparisons. Root numbers corresponding to each experimental condition are indicated at the bottom of the plot.

As PAT enables the efficient measurement of periderm length, which is an output of periderm development, we wanted to investigate whether it can distinguish mutants with defects in periderm development from wild type. For this, we used *wox4-1* mutant plants, which have a distinctive periderm phenotype, featuring reduced phellem length, altered phellem ratio, impaired auxin response, and a decreased number of periderm layers as demonstrated through cross-sectional analysis [14]. We conducted PAT analyses in 2 independent experiments using the *wox4-1* mutant line and Col-0 as a wild-type control. PAT analysis in both experiments consistently reported a significant decrease in periderm length in the *wox4-1* mutant compared to the Col-0 control (Fig. 8) (Exp 1: *P* = 3.893e-05 and Exp 2: *P* = 0.036) while no significant difference in periderm length between *wox4-1* plants in experiment 1 and experiment 2 (*P* = 0.14) or between the periderm length in Col-0 controls (*P* = 0.063) were found. However, we noted a high variability between the experiments that were conducted a week apart. This variability was most pronounced in the Col-0 genotype, which exhibited a wide range of periderm values in experiment 2 (Fig. 8). To test whether this was due to PAT, we also conducted manual measurements, which confirmed this to stem from biological variability and not from PAT inaccuracy (Fig. S5 and Table S3). Pixel values (outputted from PAT) for both Col-0 and *wox4-1* regarding periderm and root length are provided in Table S3. Overall, the ability of PAT to consistently identify the distinctive periderm phenotype in the *wox4-1* mutant supports its utility as a robust tool for characterizing and monitoring periderm development in diverse genetic backgrounds.

### Periderm length is a plastic trait

**Fig. 9.**
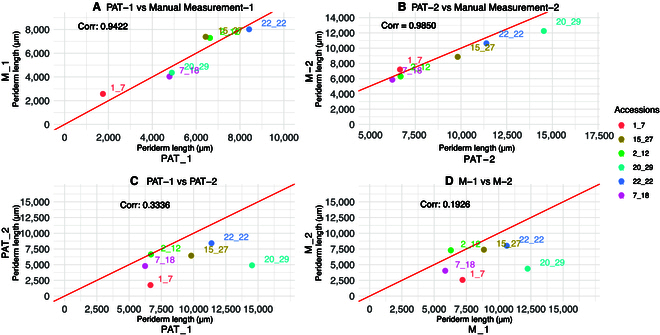
Correlations of average periderm length of 14-d-old seedlings of 6 accessions measured 36 months apart. Data from the first run (PAT-1 and M-1) is denoted by “1,” while data from the rerun/second experiment (PAT-2 and M-2) is indicated with “2.” (A) PAT-1 versus Manual Measurement-1. (B) PAT-2 versus Manual Measurement-2. (C) PAT-1 versus PAT-2. (D) M-1 versus M-2.

Given the pronounced biological variation within the Col-0 for the periderm length phenotype, we then set out to test consistency and reproducibility of periderm length measurements. For this, we regrew a randomly selected subset of 6 of the 20 examined accessions (see Materials and Methods). The seeds of these accessions were from a different seed batch as the original ones and the second experiment was conducted approximately 36 months apart. Manual and PAT measurements of this repeat were highly correlated (Fig. 9A and B). However, the correlation of periderm lengths of the two 36-months-apart repeat was only lowly to modestly correlated with 33% correlation of the PAT measurements between the 2 experiments (Fig. 9C and D). Much of the lack of correlation could be attributed to 2 of the 6 accessions, 1_7 and 20_29, as the correlation of the PAT measurements of the 36-months-apart experiments increased to 82% when omitting these 2 accessions (Fig. S7). Overall, the data show that PAT can reliably measure periderm length but that there can be substantial variation of periderm length between experiments indicating that periderm length can be a highly plastic trait. Periderm length data from both experiments (PAT and manual measurements) are provided in Table S4 and Fig. S6.

Overall, our work has shown that we can measure periderm length in an efficient and largely automated way that is suitable for genetic approaches. With this work, we have also shown for the first time that there is a notable natural variation in periderm length of Arabidopsis (Table 4). This can, in combination with PAT, be leveraged for quantitative genetic screens such as genome-wide association studies to identify the genes and their alleles underlying this natural variation.

**Table 4.** Averages of all the periderm measurements (manual and pipeline) per accession after QC

This work ID	Accession ID	Country	Periderm length (μm)
22_22	Acc-ID: 7223/Li-2:1	GER	8,135
15_27	Acc-ID: 6149/T970	Sweden	7,121
2_12	Acc-ID:5921/DraIV 3-7	Czech Republic	6,463
26_15	Acc-ID: 9382/Fri 2	Sweden	5,081
16_34	Acc-ID: 6276/TV-30	Sweden	5,056
20_29	Acc-ID: 2106/MSGA-10	US	4,460
7_18	Acc-ID:9892/IP-Sam-0	Spain	3,610
28_11	Acc-ID: 9532/IP-Cdo-0	Spain	3,470
4_18	Acc-ID:9558/IP-Moc-11	Spain	3,280
21_12	Acc-ID: 7333/Sei-0	Italy	2,669
1_7	Acc-ID:1829/Mdn-1	USA	2,187
17_23	Acc-ID: 6909/Col-0	USA	1,780
27_10	Acc-ID: 9452/Spro 3	Sweden	861
19_3	Acc-ID: 7014/Ba-1	UK	814
3_23	Acc-ID:7424/Jl-3	Czech Republic	745
25_8	Acc-ID: 8427/Ull2-13	Sweden	520
13_3	Acc-ID: 5748/Kil-0	UK	402

## Discussion

Plant secondary growth involves the periderm replacing the epidermis as the primary protective tissue for the vasculature against biotic and abiotic stresses [1,2]. The periderm originates from a series of cellular divisions in the pericycle, which leads to the development of the phellogen [22]. The phellogen, which is a secondary meristem, undergoes anticlinal and periclinal divisions during the early stages of periderm formation and then differentiates into the phelloderm and phellem in later stages [1,14]. Research has shown that periderm development occurs at different stages of root growth, revealing the mechanisms involved in its formation [2,7]. The phellem layer, which accumulates suberin during periderm development, acts as a robust barrier against water loss and shields the plant from various biotic and abiotic stresses. The suberin in the phellem can also be stained with dyes including FY [1,14,19].

Studying periderm formation promises to reveal valuable insights into plant growth and plant capacity to adapt to environmental stressors and combat pathogens, thereby illuminating important contributors to plant resilience [15]. The lack of an efficient method for characterizing periderm at a large scale has been a limiting factor for studying periderm development. To facilitate periderm measurements at an adequate scale for genetic approaches, we created PAT, a pipeline that measures periderm at a large-scale. PAT utilizes a streamlined staining protocol, automated microscopy, and a convolutional neural network to automatically measure the periderm length from the resulting images. The pipeline works by identifying the periderm from microscopy images of FY-stained roots and reports periderm and root length in both micrometers and pixels. PAT is the first pipeline to automatically detect and measure periderm from roots. We have shown that like manual measurements of phellem length [14], PAT can measure significant differences in mutants with impaired periderm development such as the *wox4-1* mutant (Fig. 8). While PAT has demonstrated effectiveness in detecting significant differences in the *wox4-1* mutant, it is important to note that PAT’s applicability to other mutants may vary. Each periderm mutant possesses unique characteristics, and generalizing findings should be approached with caution. PAT stands out as the first automated pipeline for periderm detection and measurement in roots, emphasizing its pioneering role.

To measure the performance of PAT, we first assessed how accurately PAT could distinguish the periderm from other root tissues (endoderm and lateral roots). PAT achieved 90% precision (after postprocessing) in discerning the periderm pixels from pixels of other root tissues (Table 2 and Fig. 5). We then conducted a correlation analysis between PAT and the periderm length measured by human experts and found that PAT only achieved a correlation of 79%. This prompted us to develop an efficient tool for conducting QC on the images (Fig. 3F). With this step, PAT achieved a much higher correlation to the measurements of human experts (95%). Finally, we assessed PAT’s accuracy estimating the periderm length within and between different accessions. Our analysis revealed a 94% consistency rate between manual and pipeline measurements for 17 accessions when measuring the periderm length. These results confirm accuracy and usefulness of our pipeline.

Our work involving the *wox4-1* mutant demonstrated PAT’s ability to differentiate periderm length in such mutants from the wild type (Fig. 8, Fig. S5, and Table S3). The observed variation in periderm length between 2 independent experiments underscores the high plasticity of this trait, indicating its susceptibility to environmental influences during this stage of Arabidopsis development. This was further corroborated by the lack of strong correlations of periderm length in 2 of the 6 Arabidopsis accessions that were tested 36 months apart (Fig. 9, Fig. S7, and Table S4). Overall, this suggests that PAT is a reliable tool to measure periderm length, which is indicative of differences in periderm development, but our results also indicate that care must be taken to include sufficient experimental repeats and to avoid batch effects that might otherwise confound results. Much like for other plastic traits such as root branching patterns [23], for periderm length screens, it is therefore advisable to make conditions within the screen as uniform as possible to reduce variance and to conduct follow additional experiments to validate any conclusion. Moreover, considering utilizing the periderm-length-to-root-length ratio might be helpful for increasing standardization. As we did not measure root length in the experiments from 2020 (first run), we cannot determine whether that would have explained the observed differences in 2023. Despite the observed variability, PAT consistently aligns with manual measures, highlighting its effectiveness in keeping pace with traditional measurement methods. This underscores PAT’s capacity to provide reliable insights into periderm length, offering valuable contributions to the understanding of this dynamic trait.

Taken together, PAT promises to significantly transform a labor-intensive and time-consuming process into a streamlined and efficient method, enabling researchers to obtain periderm length measurements of many roots within a few minutes. This promises to allow scientists to conduct a variety genetic screens for identifying genes or genetic variants that modulate or perturb periderm development. This includes mutant screens or genome-wide association studies via leveraging the natural variation of periderm traits across a wide range of accessions, leveraging the more than one thousand fully sequenced Arabidopsis accessions [24]. Such studies can be used to identify genetic markers associated with periderm traits, opening new possibilities for research in this area.

### Advantages and limitations

PAT is a high throughput method to conduct image-based screens on large numbers of roots for periderm length. It is easy to use as we have provided simple instructions and a comprehensive GUI for using it (available via GitHub at:

https://github.com/Salk-Harnessing-Plants-Initiative/PAT-Pipeline-for-Analysis-of-Periderm). The simple measure of periderm (phellem) length is the output of periderm development and has been shown to capture the effects of treatments or mutations on periderm development ([14]; Fig. 8B). However, periderm length is a plastic trait, and the observed variation might impede the ability to detect genotypes with subtle differences or might light to false positives. One limitation of this study is that we have not considered whether periderm differences at different points of development might be display less variation or plasticity. Moreover, as different genotypes, treatments, and growth conditions have an impact on root development and the resulting root growth rate, periderm length might sometimes show differences that are confounded due to different growth rates. A workaround for this might be to utilize the periderm-length-to-root-length ratio. This could be done by scanning the roots prior to their preparation for staining and quantifying root length from these images. As PAT is dependent on FY staining, it might be possible that mutants that are not affected in periderm formation per se but produce a form of suberin that is not stained well with FY could be detected due to their staining differences. This is a limitation, but as suberization is a hallmark of the periderm development, investigating these mutants will be worthwhile. Moreover, PAT is intended as a screening tool and as such any functional characterization that will be the result of any PAT screen should be conducted using additional experiments and more detailed characterization of effects on the periderm.

Another limitation is image quality. PAT may be unable to discern and measure the periderm in low-quality images, such as those that are blurry. This may pose a challenge when processing hundreds of samples simultaneously, as optimally focusing on some roots may cause suboptimal focusing on others, resulting in lower-quality images. In our work, we found that attempting to discern periderm from blurry images is not advisable. We employed a Keyence automated microscope for this project, but it is essential to bear in mind that the pipeline may require retraining if applied with different microscopes and/or for other species. Another aspect to consider is the quality of the staining. The root staining needs to be good for all the samples. The periderm will not be detected in understained roots. To ensure proper FY staining, it is essential to always use a freshly prepared solution of FY in lactic acid. We also found the use of a QC to be critical (Fig. 3F). The QC allows the user to preview the microscopy images to assess whether they meet the standard for PAT. Furthermore, our observations indicate that PAT may face challenges in precisely measuring periderm length when roots are positioned in close proximity to each other. To optimize accuracy, we recommend loading no more than 6 roots per genotype on the microscopy slide and ensuring appropriate spacing between them. This precautionary measure aims to enhance the reliability of periderm length measurements by minimizing potential interference or overlap between adjacent roots during the analysis process. Moreover, to ensure optimal performance, it is imperative to utilize a computer with substantial processing capabilities as the pipeline’s effectiveness relies on a computer equipped with a robust processor. To achieve faster results with PAT, we strongly advise running it on a Linux system. While PAT can function on Windows and macOS platforms, it operates significantly slower on these systems. This discrepancy in performance is attributed to the superior parallel processing capabilities offered by GPUs, which are more effectively leveraged within a Linux environment for expedited processing and analysis. Additionally, it is essential to recognize that the retraining of the pipeline may be necessary when employing different microscopes or studying other species. Consequently, this factor should be taken into consideration during the design of the experimental setup to accommodate potential adaptations of the pipeline.

To enhance the capabilities of periderm research, we developed a pipeline to automatically measure the periderm/phellem length. This pipeline uses images that are generated by automatic microscopy of roots previously stained with FY to outline the cell contour. Overall, our image analysis pipeline is a valuable tool that can help us automate periderm length measurements in Arabidopsis roots opening new avenues of periderm research and providing an example of how large-scale biometric studies of cell types and tissues could be designed. However, care must be taken to include sufficient repeats and controls, to minimize variation, and to ensure comparability of results.

## Data Availability

All raw data and datasets have been included in the Supplementary Materials. The PAT pipeline and its associated code are accessible via the following GitHub repository: https://github.com/Salk-Harnessing-Plants-Initiative/PAT-Pipeline-for-Analysis-of-Periderm. Additionally, the test dataset comprising Col-0 and *wox4-1* TIFF images is available on the same GitHub repository. Full-resolution TIFF images corresponding to the natural accessions (Table 1) can be obtained from the corresponding author upon request.
