# Supplementary material for: PAT (Periderm Assessment Toolkit): A Quantitative and Large-Scale Screening Method for Periderm Measurements
Source: Plant Phenomics. 2024 Mar 29;6:0156. doi: 10.34133/plantphenomics.0156 (PMC10981931; doi:10.34133/plantphenomics.0156)
Supplement: Supplementary 1 — Figs. S1 to S7 Tables S1 to S4 [file plantphenomics.0156.f1.zip › Supplementary Material.docx]

**Supplementary Material**

**Fig. S1**. Post-processing of images. (A) Gaps produced due to segmentation errors are filled. If the gap is smaller or equal to 150 pixels (e.g. gap1), it is added to the continuous periderm. If the gap is bigger than 150 pixels (e.g. gap2), the predicted periderm is considered a false positive and discarded. The post-processed images contain only one continuous periderm along the main root (B) Root number is indicated in white next to the hypocotyl.

**Fig. S2**. Quality Control (QC) assessment process for microscopy images. Owing to the elongated nature of the roots, displaying all details without zooming in was not feasible. Therefore, we cropped 1500 pixels on either side (left and right, marked by a red box) of the transition point from periderm to endodermis for each root. A graphical user interface (GUI) is provided to the user to view the image and determine if it meets the minimum quality required for further processing. This QC process also enables the user to identify specific roots that may be blurry. The GUI displays a periderm portion of the root stained with FY, and a suggested pipeline segmentation represented by a light blue color. The first root shown meets the standard for the pipeline, indicating that it is not blurry, and the periderm segmentation is accurate. The user selects this root and moves on to the next one. The good quality-selected-images are then run in PAT. Light blue indicates periderm, and dark blue indicates endoderm segmentation.

**Fig. S3**. Representative image of the blurred upper periderm portion below the hypocotyl in the roots from accessions 5_24, 23_12, and 18_8.

**Fig. S4.** TIFF images corresponding to Col-0 (wild-type) and the wox4 mutant obtained from two independent experiments, designated as "Rep. 1" and "Rep. 2." These images have been provided to enable users to assess and validate the functionality and reliability of the PAT pipeline for periderm analysis.

Col_rep1.


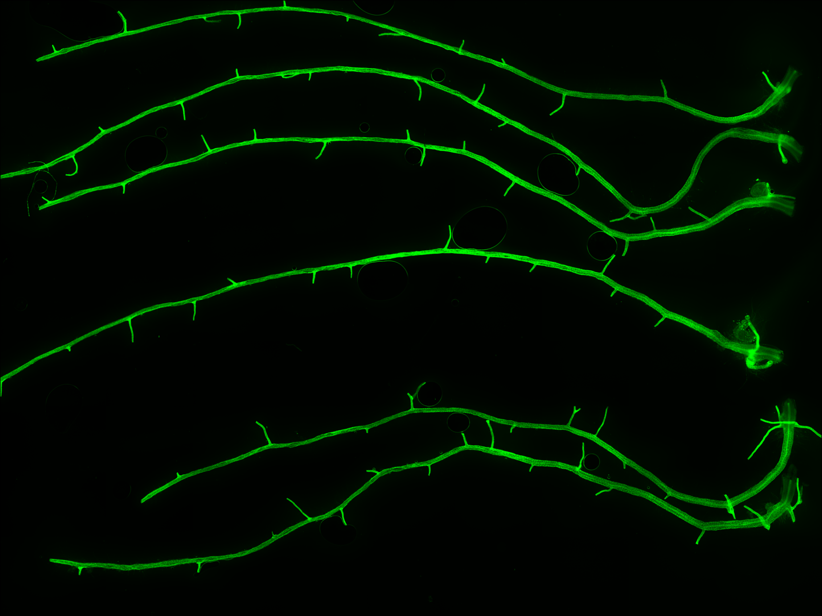


Col_rep2


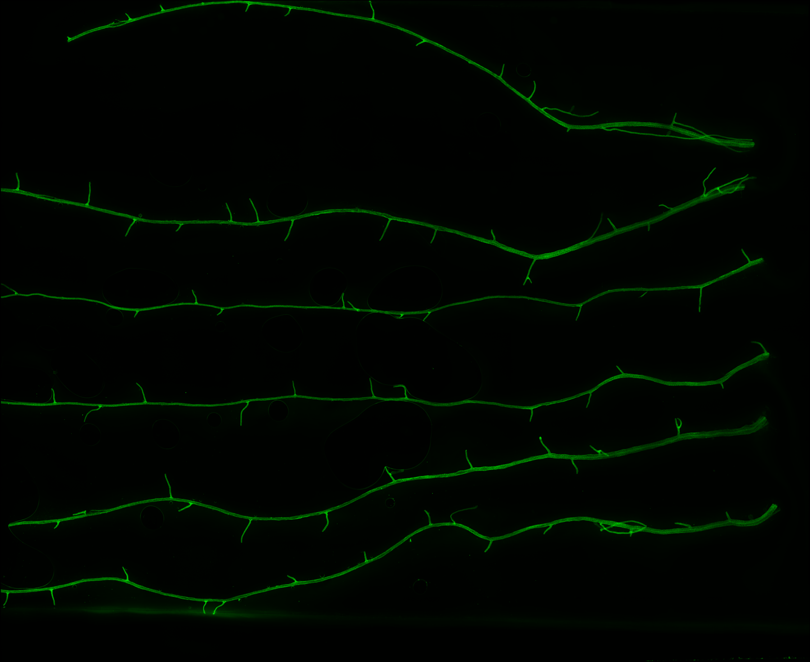


WOX4_rep1


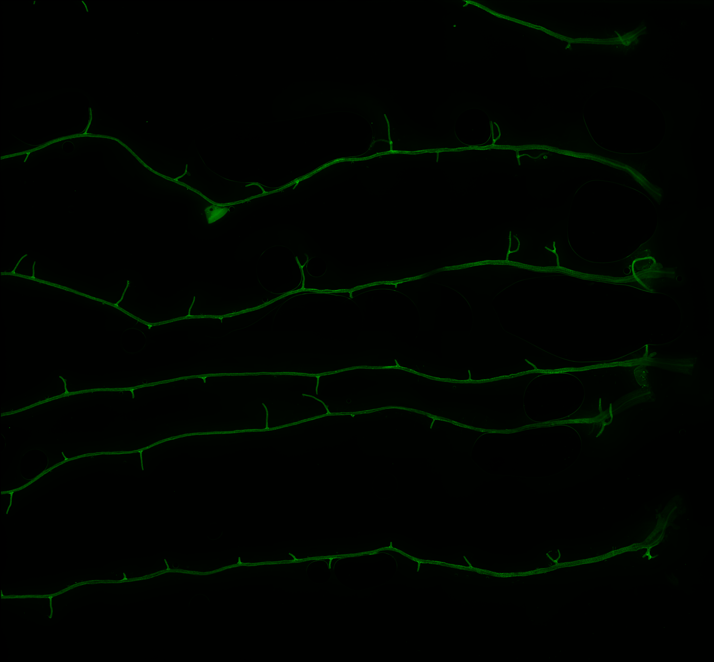


WOX4_rep2

**
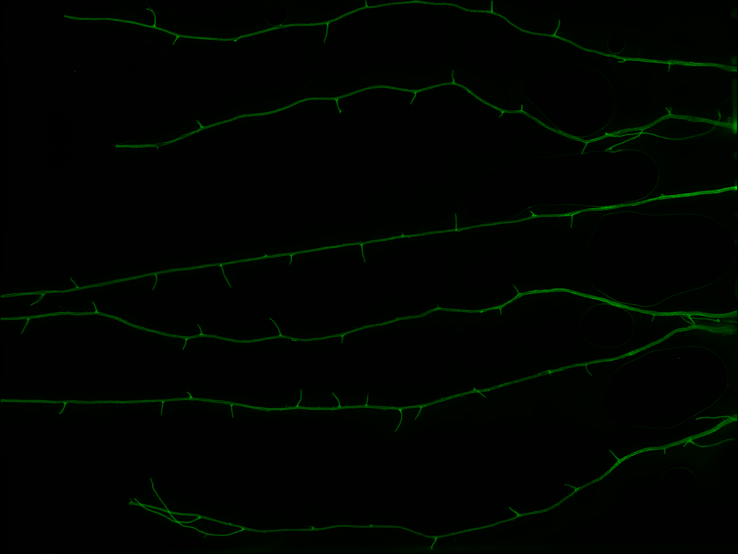
**

**Fig. S5.** Two independent experiments, labeled as "Exp.1" and "Exp.2," were conducted, with Exp.2 performed one week later than Exp.1. Data obtained from the PAT pipeline is denoted as PAT, while data from manual measurements is labeled as M. (A) Comparison of *wox4-1* and Col-0 root periderm measurements (in μm). (B) Comparison of *wox4-1* and Col-0 root length measurements (in μm). (C) Periderm/root length ratio (calculated by dividing the length of the periderm by the length of the root). Two-sided t-test P-values for each comparison is provided.

**
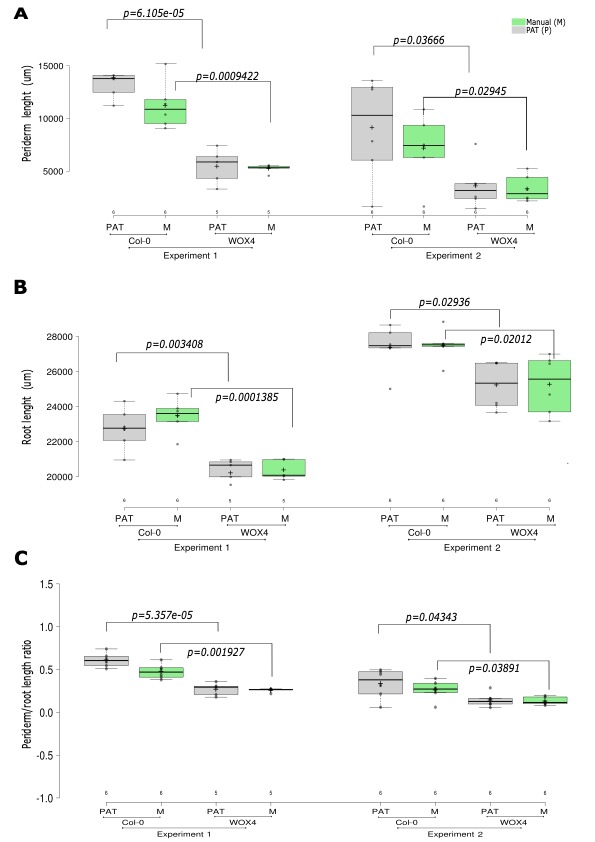
**

**Fig S6.** Reproducibility Experiments of Periderm Length Measurements using the PAT Pipeline. Periderm length measurements (in micrometers) for six randomly selected accessions (1_7, 2_12, 7_18, 15_27, 20_29, and 22_22) were obtained using the PAT pipeline (P) and manual measurements (M). The original results are presented as "1st run" (using subscript 1 for both P and M), and the re-run experiment is indicated as "2nd run" (using subscript 2 for both P and M) approximately 36 months later. The P-value (p) from the two-sided t-test is provided, with NS denoting non-significant differences in periderm length.


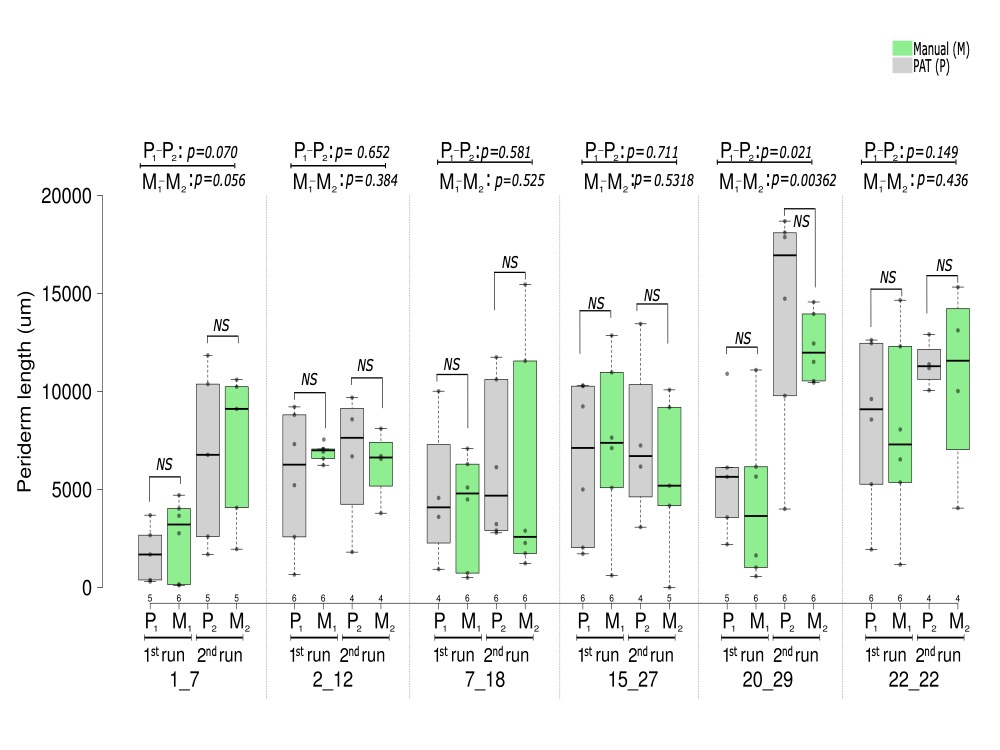


**Fig. S7.** Correlations of average periderm length of 14-day old seedlings of 6 accessions measured 36 months apart with outlier accessions removed. Data from the 1st run (PAT-1 and M-1) is denoted by "1," while data from the re-run/2^nd^ experiment (PAT-2 and M-2) is indicated with "2." (A) PAT-1 vs Manual Measurement-1 (B) PAT-2 vs Manual Measurement-2 (C) PAT-1 vs PAT-2 (D) M-1 vs M-2 . Accessions 1_7 and 20_29 were removed from the analysis.

**Table S1**. Periderm length values in micrometers for all the 20 accessions utilized in this study before the quality control (QC) process was applied. PAT = measurements obtained via an automatic pipeline, M1 = manual measurements from the first individual, M2 = manual measurements from the second individual.

| Samples | Benchmark | Periderm length (um) |
| --- | --- | --- |
| 1_7 | PAT | 1685 |
| 1_7 | PAT | 3692 |
| 1_7 | PAT | 306 |
| 1_7 | PAT | 2671 |
| 1_7 | PAT | 0 |
| 1_7 | PAT | 387 |
| 1_7 | M1 | 4712 |
| 1_7 | M1 | 4028 |
| 1_7 | M1 | 160 |
| 1_7 | M1 | 3663 |
| 1_7 | M1 | 2766 |
| 1_7 | M1 | 111 |
| 1_7 | M2 | 4509 |
| 1_7 | M2 | 3902 |
| 1_7 | M2 | 218 |
| 1_7 | M2 | 3616 |
| 1_7 | M2 | 2632 |
| 1_7 | M2 | 317 |
| 2_12 | PAT | 7316 |
| 2_12 | PAT | 659 |
| 2_12 | PAT | 5221 |
| 2_12 | PAT | 8810 |
| 2_12 | PAT | 9216 |
| 2_12 | PAT | 2585 |
| 2_12 | M1 | 6934 |
| 2_12 | M1 | 6582 |
| 2_12 | M1 | 7066 |
| 2_12 | M1 | 6240 |
| 2_12 | M1 | 7066 |
| 2_12 | M1 | 9850 |
| 2_12 | M2 | 6791 |
| 2_12 | M2 | 6885 |
| 2_12 | M2 | 6908 |
| 2_12 | M2 | 6204 |
| 2_12 | M2 | 6572 |
| 2_12 | M2 | 5431 |
| 3_23 | PAT | 0 |
| 3_23 | PAT | 0 |
| 3_23 | PAT | 3640 |
| 3_23 | PAT | 0 |
| 3_23 | PAT | 1261 |
| 3_23 | PAT | 0 |
| 3_23 | M1 | 2447 |
| 3_23 | M1 | 0 |
| 3_23 | M1 | 566 |
| 3_23 | M1 | 1517 |
| 3_23 | M1 | 0 |
| 3_23 | M1 | 887 |
| 3_23 | M2 | 1557 |
| 3_23 | M2 | 0 |
| 3_23 | M2 | 406 |
| 3_23 | M2 | 350 |
| 3_23 | M2 | 792 |
| 3_23 | M2 | 0 |
| 4_18 | PAT | 3081 |
| 4_18 | PAT | 6236 |
| 4_18 | PAT | 2531 |
| 4_18 | PAT | 2325 |
| 4_18 | PAT | 3404 |
| 4_18 | PAT | 2872 |
| 4_18 | M1 | 3983 |
| 4_18 | M1 | 5044 |
| 4_18 | M1 | 1560 |
| 4_18 | M1 | 2685 |
| 4_18 | M1 | 3541 |
| 4_18 | M1 | 3110 |
| 4_18 | M2 | 3336 |
| 4_18 | M2 | 5921 |
| 4_18 | M2 | 1127 |
| 4_18 | M2 | 2806 |
| 4_18 | M2 | 3003 |
| 4_18 | M2 | 2484 |
| 5_24 | PAT | 11292 |
| 5_24 | PAT | 12857 |
| 5_24 | PAT | 10353 |
| 5_24 | PAT | 8977 |
| 5_24 | M1 | 5964 |
| 5_24 | M1 | 8605 |
| 5_24 | M1 | 5507 |
| 5_24 | M1 | 5408 |
| 5_24 | M1 | 7077 |
| 5_24 | M1 | 2928 |
| 5_24 | M2 | 5637 |
| 5_24 | M2 | 8416 |
| 5_24 | M2 | 3987 |
| 5_24 | M2 | 5245 |
| 5_24 | M2 | 8343 |
| 5_24 | M2 | 2333 |
| 7_18 | PAT | 4573 |
| 7_18 | PAT | 10017 |
| 7_18 | PAT | 3608 |
| 7_18 | PAT | 0 |
| 7_18 | PAT | 0 |
| 7_18 | PAT | 933 |
| 7_18 | M1 | 6031 |
| 7_18 | M1 | 7096 |
| 7_18 | M1 | 6229 |
| 7_18 | M1 | 659 |
| 7_18 | M1 | 1093 |
| 7_18 | M1 | 527 |
| 7_18 | M2 | 6294 |
| 7_18 | M2 | 7090 |
| 7_18 | M2 | 5102 |
| 7_18 | M2 | 4494 |
| 7_18 | M2 | 736 |
| 7_18 | M2 | 500 |
| 13_3 | PAT | 0 |
| 13_3 | PAT | 571 |
| 13_3 | PAT | 1434 |
| 13_3 | PAT | 999 |
| 13_3 | PAT | 780 |
| 13_3 | PAT | 563 |
| 13_3 | M1 | 566 |
| 13_3 | M1 | 337 |
| 13_3 | M1 | 240 |
| 13_3 | M1 | 69 |
| 13_3 | M1 | 0 |
| 13_3 | M1 | 0 |
| 13_3 | M2 | 334 |
| 13_3 | M2 | 435 |
| 13_3 | M2 | 312 |
| 13_3 | M2 | 98 |
| 13_3 | M2 | 61 |
| 13_3 | M2 | 40 |
| 15_27 | PAT | 9241 |
| 15_27 | PAT | 5002 |
| 15_27 | PAT | 10273 |
| 15_27 | PAT | 1718 |
| 15_27 | PAT | 2039 |
| 15_27 | PAT | 10318 |
| 15_27 | M1 | 9788 |
| 15_27 | M1 | 5965 |
| 15_27 | M1 | 12437 |
| 15_27 | M1 | 6152 |
| 15_27 | M1 | 1585 |
| 15_27 | M1 | 9357 |
| 15_27 | M2 | 10976 |
| 15_27 | M2 | 5092 |
| 15_27 | M2 | 12860 |
| 15_27 | M2 | 7648 |
| 15_27 | M2 | 616 |
| 15_27 | M2 | 7116 |
| 16_34 | PAT | 3220 |
| 16_34 | PAT | 8473 |
| 16_34 | PAT | 1457 |
| 16_34 | PAT | 4516 |
| 16_34 | PAT | 821 |
| 16_34 | PAT | 5667 |
| 16_34 | M1 | 3861 |
| 16_34 | M1 | 7376 |
| 16_34 | M1 | 6233 |
| 16_34 | M1 | 1768 |
| 16_34 | M1 | 7714 |
| 16_34 | M1 | 4126 |
| 16_34 | M2 | 3246 |
| 16_34 | M2 | 8866 |
| 16_34 | M2 | 6975 |
| 16_34 | M2 | 2330 |
| 16_34 | M2 | 9720 |
| 16_34 | M2 | 4647 |
| 17_23 | PAT | 4466 |
| 17_23 | PAT | 3155 |
| 17_23 | PAT | 1109 |
| 17_23 | PAT | 852 |
| 17_23 | PAT | 2149 |
| 17_23 | PAT | 741 |
| 17_23 | M1 | 4098 |
| 17_23 | M1 | 2729 |
| 17_23 | M1 | 1524 |
| 17_23 | M1 | 218 |
| 17_23 | M1 | 1633 |
| 17_23 | M1 | 705 |
| 17_23 | M2 | 3581 |
| 17_23 | M2 | 2778 |
| 17_23 | M2 | 851 |
| 17_23 | M2 | 253 |
| 17_23 | M2 | 798 |
| 17_23 | M2 | 414 |
| 18_8 | PAT | 7102 |
| 18_8 | PAT | 0 |
| 18_8 | PAT | 4338 |
| 18_8 | PAT | 5822 |
| 18_8 | PAT | 2279 |
| 18_8 | PAT | 7009 |
| 18_8 | M1 | 1476 |
| 18_8 | M1 | 1231 |
| 18_8 | M1 | 2503 |
| 18_8 | M1 | 1211 |
| 18_8 | M1 | 0 |
| 18_8 | M1 | 3746 |
| 18_8 | M2 | 1500 |
| 18_8 | M2 | 1144 |
| 18_8 | M2 | 2005 |
| 18_8 | M2 | 1360 |
| 18_8 | M2 | 190 |
| 18_8 | M2 | 3967 |
| 19_3 | PAT | 744 |
| 19_3 | PAT | 2200 |
| 19_3 | PAT | 1148 |
| 19_3 | PAT | 1038 |
| 19_3 | PAT | 1076 |
| 19_3 | M1 | 407 |
| 19_3 | M1 | 685 |
| 19_3 | M1 | 700 |
| 19_3 | M1 | 716 |
| 19_3 | M1 | 409 |
| 19_3 | M2 | 649 |
| 19_3 | M2 | 586 |
| 19_3 | M2 | 875 |
| 19_3 | M2 | 549 |
| 19_3 | M2 | 428 |
| 20_29 | PAT | 11219 |
| 20_29 | PAT | 0 |
| 20_29 | PAT | 6121 |
| 20_29 | PAT | 2201 |
| 20_29 | PAT | 5647 |
| 20_29 | PAT | 3583 |
| 20_29 | M1 | 12249 |
| 20_29 | M1 | 51 |
| 20_29 | M1 | 5309 |
| 20_29 | M1 | 1101 |
| 20_29 | M1 | 5630 |
| 20_29 | M1 | 1031 |
| 20_29 | M2 | 11099 |
| 20_29 | M2 | 571 |
| 20_29 | M2 | 6161 |
| 20_29 | M2 | 1640 |
| 20_29 | M2 | 5660 |
| 20_29 | M2 | 1024 |
| 21_12 | PAT | 7409 |
| 21_12 | PAT | 685 |
| 21_12 | PAT | 1807 |
| 21_12 | PAT | 721 |
| 21_12 | PAT | 6470 |
| 21_12 | PAT | 777 |
| 21_12 | M1 | 4437 |
| 21_12 | M1 | 250 |
| 21_12 | M1 | 2425 |
| 21_12 | M1 | 4034 |
| 21_12 | M1 | 1976 |
| 21_12 | M1 | 3398 |
| 21_12 | M2 | 4059 |
| 21_12 | M2 | 220 |
| 21_12 | M2 | 2135 |
| 21_12 | M2 | 2098 |
| 21_12 | M2 | 2091 |
| 21_12 | M2 | 3054 |
| 22_22 | PAT | 9617 |
| 22_22 | PAT | 5271 |
| 22_22 | PAT | 8570 |
| 22_22 | PAT | 12463 |
| 22_22 | PAT | 12627 |
| 22_22 | PAT | 1941 |
| 22_22 | M1 | 13747 |
| 22_22 | M1 | 5867 |
| 22_22 | M1 | 7634 |
| 22_22 | M1 | 6909 |
| 22_22 | M1 | 12263 |
| 22_22 | M1 | 1435 |
| 22_22 | M2 | 12301 |
| 22_22 | M2 | 5364 |
| 22_22 | M2 | 8064 |
| 22_22 | M2 | 6535 |
| 22_22 | M2 | 14659 |
| 22_22 | M2 | 1168 |
| 23_12 | PAT | 1862 |
| 23_12 | PAT | 4372 |
| 23_12 | PAT | 11477 |
| 23_12 | PAT | 4083 |
| 23_12 | PAT | 3566 |
| 23_12 | PAT | 507 |
| 23_12 | M1 | 1268 |
| 23_12 | M1 | 725 |
| 23_12 | M1 | 410 |
| 23_12 | M1 | 0 |
| 23_12 | M1 | 1223 |
| 23_12 | M1 | 0 |
| 23_12 | M2 | 1405 |
| 23_12 | M2 | 274 |
| 23_12 | M2 | 910 |
| 23_12 | M2 | 0 |
| 23_12 | M2 | 0 |
| 23_12 | M2 | 0 |
| 25_8 | PAT | 470 |
| 25_8 | PAT | 2867 |
| 25_8 | PAT | 470 |
| 25_8 | PAT | 192 |
| 25_8 | PAT | 1003 |
| 25_8 | M1 | 255 |
| 25_8 | M1 | 1024 |
| 25_8 | M1 | 0 |
| 25_8 | M1 | 0 |
| 25_8 | M1 | 0 |
| 25_8 | M2 | 381 |
| 25_8 | M2 | 1145 |
| 25_8 | M2 | 0 |
| 25_8 | M2 | 0 |
| 25_8 | M2 | 0 |
| 26_15 | PAT | 5097 |
| 26_15 | PAT | 4248 |
| 26_15 | PAT | 7395 |
| 26_15 | PAT | 9124 |
| 26_15 | PAT | 4064 |
| 26_15 | PAT | 228 |
| 26_15 | M1 | 5497 |
| 26_15 | M1 | 3920 |
| 26_15 | M1 | 6416 |
| 26_15 | M1 | 9584 |
| 26_15 | M1 | 3392 |
| 26_15 | M1 | 1820 |
| 26_15 | M2 | 5388 |
| 26_15 | M2 | 3347 |
| 26_15 | M2 | 6343 |
| 26_15 | M2 | 9077 |
| 26_15 | M2 | 4452 |
| 26_15 | M2 | 2078 |
| 27_10 | PAT | 538 |
| 27_10 | PAT | 755 |
| 27_10 | PAT | 0 |
| 27_10 | PAT | 0 |
| 27_10 | PAT | 876 |
| 27_10 | M1 | 937 |
| 27_10 | M1 | 2203 |
| 27_10 | M1 | 0 |
| 27_10 | M1 | 0 |
| 27_10 | M1 | 2088 |
| 27_10 | M2 | 781 |
| 27_10 | M2 | 2278 |
| 27_10 | M2 | 0 |
| 27_10 | M2 | 0 |
| 27_10 | M2 | 2462 |
| 28_11 | PAT | 3128 |
| 28_11 | PAT | 3165 |
| 28_11 | PAT | 8503 |
| 28_11 | PAT | 3394 |
| 28_11 | PAT | 778 |
| 28_11 | PAT | 3491 |
| 28_11 | M1 | 3104 |
| 28_11 | M1 | 2746 |
| 28_11 | M1 | 7980 |
| 28_11 | M1 | 3192 |
| 28_11 | M1 | 0 |
| 28_11 | M1 | 2202 |
| 28_11 | M2 | 3224 |
| 28_11 | M2 | 2970 |
| 28_11 | M2 | 7747 |
| 28_11 | M2 | 3830 |
| 28_11 | M2 | 458 |
| 28_11 | M2 | 2563 |

**Table S2.** Periderm length values in micrometers for all accessions in the study after undergoing the quality control (QC) process. PAT = measurements obtained via an automatic pipeline, M1 = manual measurements from the first individual, and M2 = manual measurements from the second individual. Accessions 5_24, 23_12, and 18_8 were excluded from the study due to the blurriness of their images identified during QC.

| Samples | Benchmark | Periderm_length (um) |
| --- | --- | --- |
| 1_7 | PAT | 1685 |
| 1_7 | PAT | 3692 |
| 1_7 | PAT | 306 |
| 1_7 | PAT | 2671 |
| 1_7 | PAT | 0 |
| 1_7 | PAT | 387 |
| 1_7 | M1 | 4712 |
| 1_7 | M1 | 4028 |
| 1_7 | M1 | 160 |
| 1_7 | M1 | 3663 |
| 1_7 | M1 | 2766 |
| 1_7 | M1 | 111 |
| 1_7 | M2 | 4509 |
| 1_7 | M2 | 3902 |
| 1_7 | M2 | 218 |
| 1_7 | M2 | 3616 |
| 1_7 | M2 | 2632 |
| 1_7 | M2 | 317 |
| 2_12 | PAT | 7316 |
| 2_12 | PAT | 659 |
| 2_12 | PAT | 5221 |
| 2_12 | PAT | 8810 |
| 2_12 | PAT | 9216 |
| 2_12 | PAT | 2585 |
| 2_12 | M1 | 6934 |
| 2_12 | M1 | 6582 |
| 2_12 | M1 | 7066 |
| 2_12 | M1 | 6240 |
| 2_12 | M1 | 7066 |
| 2_12 | M1 | 9850 |
| 2_12 | M2 | 6791 |
| 2_12 | M2 | 6885 |
| 2_12 | M2 | 6908 |
| 2_12 | M2 | 6204 |
| 2_12 | M2 | 6572 |
| 2_12 | M2 | 5431 |
| 3_23 | PAT | 0 |
| 3_23 | PAT | 0 |
| 3_23 | PAT | 3640 |
| 3_23 | PAT | 0 |
| 3_23 | PAT | 1261 |
| 3_23 | PAT | 0 |
| 3_23 | M1 | 2447 |
| 3_23 | M1 | 0 |
| 3_23 | M1 | 566 |
| 3_23 | M1 | 1517 |
| 3_23 | M1 | 0 |
| 3_23 | M1 | 887 |
| 3_23 | M2 | 1557 |
| 3_23 | M2 | 0 |
| 3_23 | M2 | 406 |
| 3_23 | M2 | 350 |
| 3_23 | M2 | 792 |
| 3_23 | M2 | 0 |
| 4_18 | PAT | 3081 |
| 4_18 | PAT | 6236 |
| 4_18 | PAT | 2531 |
| 4_18 | PAT | 2325 |
| 4_18 | PAT | 3404 |
| 4_18 | PAT | 2872 |
| 4_18 | M1 | 3983 |
| 4_18 | M1 | 5044 |
| 4_18 | M1 | 1560 |
| 4_18 | M1 | 2685 |
| 4_18 | M1 | 3541 |
| 4_18 | M1 | 3110 |
| 4_18 | M2 | 3336 |
| 4_18 | M2 | 5921 |
| 4_18 | M2 | 1127 |
| 4_18 | M2 | 2806 |
| 4_18 | M2 | 3003 |
| 4_18 | M2 | 2484 |
| 7_18 | PAT | 4573 |
| 7_18 | PAT | 10017 |
| 7_18 | PAT | 3608 |
| 7_18 | PAT | 0 |
| 7_18 | PAT | 0 |
| 7_18 | PAT | 933 |
| 7_18 | M1 | 6031 |
| 7_18 | M1 | 7096 |
| 7_18 | M1 | 6229 |
| 7_18 | M1 | 659 |
| 7_18 | M1 | 1093 |
| 7_18 | M1 | 527 |
| 7_18 | M2 | 6294 |
| 7_18 | M2 | 7090 |
| 7_18 | M2 | 5102 |
| 7_18 | M2 | 4494 |
| 7_18 | M2 | 736 |
| 7_18 | M2 | 500 |
| 13_3 | PAT | 0 |
| 13_3 | PAT | 571 |
| 13_3 | PAT | 1434 |
| 13_3 | PAT | 999 |
| 13_3 | PAT | 780 |
| 13_3 | PAT | 563 |
| 13_3 | M1 | 566 |
| 13_3 | M1 | 337 |
| 13_3 | M1 | 240 |
| 13_3 | M1 | 69 |
| 13_3 | M1 | 0 |
| 13_3 | M1 | 0 |
| 13_3 | M2 | 334 |
| 13_3 | M2 | 435 |
| 13_3 | M2 | 312 |
| 13_3 | M2 | 98 |
| 13_3 | M2 | 61 |
| 13_3 | M2 | 40 |
| 15_27 | PAT | 9241 |
| 15_27 | PAT | 5002 |
| 15_27 | PAT | 10273 |
| 15_27 | PAT | 1718 |
| 15_27 | PAT | 2039 |
| 15_27 | PAT | 10318 |
| 15_27 | M1 | 9788 |
| 15_27 | M1 | 5965 |
| 15_27 | M1 | 12437 |
| 15_27 | M1 | 6152 |
| 15_27 | M1 | 1585 |
| 15_27 | M1 | 9357 |
| 15_27 | M2 | 10976 |
| 15_27 | M2 | 5092 |
| 15_27 | M2 | 12860 |
| 15_27 | M2 | 7648 |
| 15_27 | M2 | 616 |
| 15_27 | M2 | 7116 |
| 16_34 | PAT | 3220 |
| 16_34 | PAT | 8473 |
| 16_34 | PAT | 1457 |
| 16_34 | PAT | 4516 |
| 16_34 | PAT | 821 |
| 16_34 | PAT | 5667 |
| 16_34 | M1 | 3861 |
| 16_34 | M1 | 7376 |
| 16_34 | M1 | 6233 |
| 16_34 | M1 | 1768 |
| 16_34 | M1 | 7714 |
| 16_34 | M1 | 4126 |
| 16_34 | M2 | 3246 |
| 16_34 | M2 | 8866 |
| 16_34 | M2 | 6975 |
| 16_34 | M2 | 2330 |
| 16_34 | M2 | 9720 |
| 16_34 | M2 | 4647 |
| 17_23 | PAT | 4466 |
| 17_23 | PAT | 3155 |
| 17_23 | PAT | 1109 |
| 17_23 | PAT | 852 |
| 17_23 | PAT | 2149 |
| 17_23 | PAT | 741 |
| 17_23 | M1 | 4098 |
| 17_23 | M1 | 2729 |
| 17_23 | M1 | 1524 |
| 17_23 | M1 | 218 |
| 17_23 | M1 | 1633 |
| 17_23 | M1 | 705 |
| 17_23 | M2 | 3581 |
| 17_23 | M2 | 2778 |
| 17_23 | M2 | 851 |
| 17_23 | M2 | 253 |
| 17_23 | M2 | 798 |
| 17_23 | M2 | 414 |
| 19_3 | PAT | 744 |
| 19_3 | PAT | 2200 |
| 19_3 | PAT | 1148 |
| 19_3 | PAT | 1038 |
| 19_3 | PAT | 1076 |
| 19_3 | M1 | 407 |
| 19_3 | M1 | 685 |
| 19_3 | M1 | 700 |
| 19_3 | M1 | 716 |
| 19_3 | M1 | 409 |
| 19_3 | M2 | 649 |
| 19_3 | M2 | 586 |
| 19_3 | M2 | 875 |
| 19_3 | M2 | 549 |
| 19_3 | M2 | 428 |
| 20_29 | PAT | 11219 |
| 20_29 | PAT | 0 |
| 20_29 | PAT | 6121 |
| 20_29 | PAT | 2201 |
| 20_29 | PAT | 5647 |
| 20_29 | PAT | 3583 |
| 20_29 | M1 | 12249 |
| 20_29 | M1 | 51 |
| 20_29 | M1 | 5309 |
| 20_29 | M1 | 1101 |
| 20_29 | M1 | 5630 |
| 20_29 | M1 | 1031 |
| 20_29 | M2 | 11099 |
| 20_29 | M2 | 571 |
| 20_29 | M2 | 6161 |
| 20_29 | M2 | 1640 |
| 20_29 | M2 | 5660 |
| 20_29 | M2 | 1024 |
| 21_12 | PAT | 7409 |
| 21_12 | PAT | 685 |
| 21_12 | PAT | 1807 |
| 21_12 | PAT | 721 |
| 21_12 | PAT | 6470 |
| 21_12 | PAT | 777 |
| 21_12 | M1 | 4437 |
| 21_12 | M1 | 250 |
| 21_12 | M1 | 2425 |
| 21_12 | M1 | 4034 |
| 21_12 | M1 | 1976 |
| 21_12 | M1 | 3398 |
| 21_12 | M2 | 4059 |
| 21_12 | M2 | 220 |
| 21_12 | M2 | 2135 |
| 21_12 | M2 | 2098 |
| 21_12 | M2 | 2091 |
| 21_12 | M2 | 3054 |
| 22_22 | PAT | 9617 |
| 22_22 | PAT | 5271 |
| 22_22 | PAT | 8570 |
| 22_22 | PAT | 12463 |
| 22_22 | PAT | 12627 |
| 22_22 | PAT | 1941 |
| 22_22 | M1 | 13747 |
| 22_22 | M1 | 5867 |
| 22_22 | M1 | 7634 |
| 22_22 | M1 | 6909 |
| 22_22 | M1 | 12263 |
| 22_22 | M1 | 1435 |
| 22_22 | M2 | 12301 |
| 22_22 | M2 | 5364 |
| 22_22 | M2 | 8064 |
| 22_22 | M2 | 6535 |
| 22_22 | M2 | 14659 |
| 22_22 | M2 | 1168 |
| 25_8 | PAT | 470 |
| 25_8 | PAT | 2867 |
| 25_8 | PAT | 470 |
| 25_8 | PAT | 192 |
| 25_8 | PAT | 1003 |
| 25_8 | M1 | 255 |
| 25_8 | M1 | 1024 |
| 25_8 | M1 | 0 |
| 25_8 | M1 | 0 |
| 25_8 | M1 | 0 |
| 25_8 | M2 | 381 |
| 25_8 | M2 | 1145 |
| 25_8 | M2 | 0 |
| 25_8 | M2 | 0 |
| 25_8 | M2 | 0 |
| 26_15 | PAT | 5097 |
| 26_15 | PAT | 4248 |
| 26_15 | PAT | 7395 |
| 26_15 | PAT | 9124 |
| 26_15 | PAT | 4064 |
| 26_15 | PAT | 228 |
| 26_15 | M1 | 5497 |
| 26_15 | M1 | 3920 |
| 26_15 | M1 | 6416 |
| 26_15 | M1 | 9584 |
| 26_15 | M1 | 3392 |
| 26_15 | M1 | 1820 |
| 26_15 | M2 | 5388 |
| 26_15 | M2 | 3347 |
| 26_15 | M2 | 6343 |
| 26_15 | M2 | 9077 |
| 26_15 | M2 | 4452 |
| 26_15 | M2 | 2078 |
| 27_10 | PAT | 538 |
| 27_10 | PAT | 755 |
| 27_10 | PAT | 0 |
| 27_10 | PAT | 0 |
| 27_10 | PAT | 876 |
| 27_10 | M1 | 937 |
| 27_10 | M1 | 2203 |
| 27_10 | M1 | 0 |
| 27_10 | M1 | 0 |
| 27_10 | M1 | 2088 |
| 27_10 | M2 | 781 |
| 27_10 | M2 | 2278 |
| 27_10 | M2 | 0 |
| 27_10 | M2 | 0 |
| 27_10 | M2 | 2462 |
| 28_11 | PAT | 3128 |
| 28_11 | PAT | 3165 |
| 28_11 | PAT | 8503 |
| 28_11 | PAT | 3394 |
| 28_11 | PAT | 778 |
| 28_11 | PAT | 3491 |
| 28_11 | M1 | 3104 |
| 28_11 | M1 | 2746 |
| 28_11 | M1 | 7980 |
| 28_11 | M1 | 3192 |
| 28_11 | M1 | 0 |
| 28_11 | M1 | 2202 |
| 28_11 | M2 | 3224 |
| 28_11 | M2 | 2970 |
| 28_11 | M2 | 7747 |
| 28_11 | M2 | 3830 |
| 28_11 | M2 | 458 |
| 28_11 | M2 | 2563 |

**Table S3.** Periderm length (μm), root length (μm) and periderm/root length ratio obtained from the *wox4-1* mutant and Col-0 across two independent experiments (Exp1 and Exp2). BQ=Bad quality images that cannot be processed. The periderm/root length ratio was calculated by dividing the length of the periderm by the length of the root. Additionally, PAT provides periderm length and root length measured in pixels.

**Table S4**. Reproducibility Experiment for PAT: Periderm Lengths (in micrometers) across Six Subset Accessions (1_7, 2_12, 7_18, 15_27, 20_29, and 22_22). The table includes original measurements from the PAT pipeline (PAT-1), manual measurements (M-1), and a re-run (2^nd^) experiment conducted ~36 months later, with corresponding measurements labeled PAT-2 and M-2. Two-sided t-test P-values for each comparison (PAT-1 vs M-1, PAT-2 vs M-2, PAT-1 vs PAT-2, M-1 vs M-2) are provided below the respective values. BQ indicates Bad Quality images.
