# Supplementary figures and images for: PAT (Periderm Assessment Toolkit): A Quantitative and Large-Scale Screening Method for Periderm Measurements
Source: Plant Phenomics. 2024 Mar 29;6:0156. doi: 10.34133/plantphenomics.0156 (PMC10981931; doi:10.34133/plantphenomics.0156)

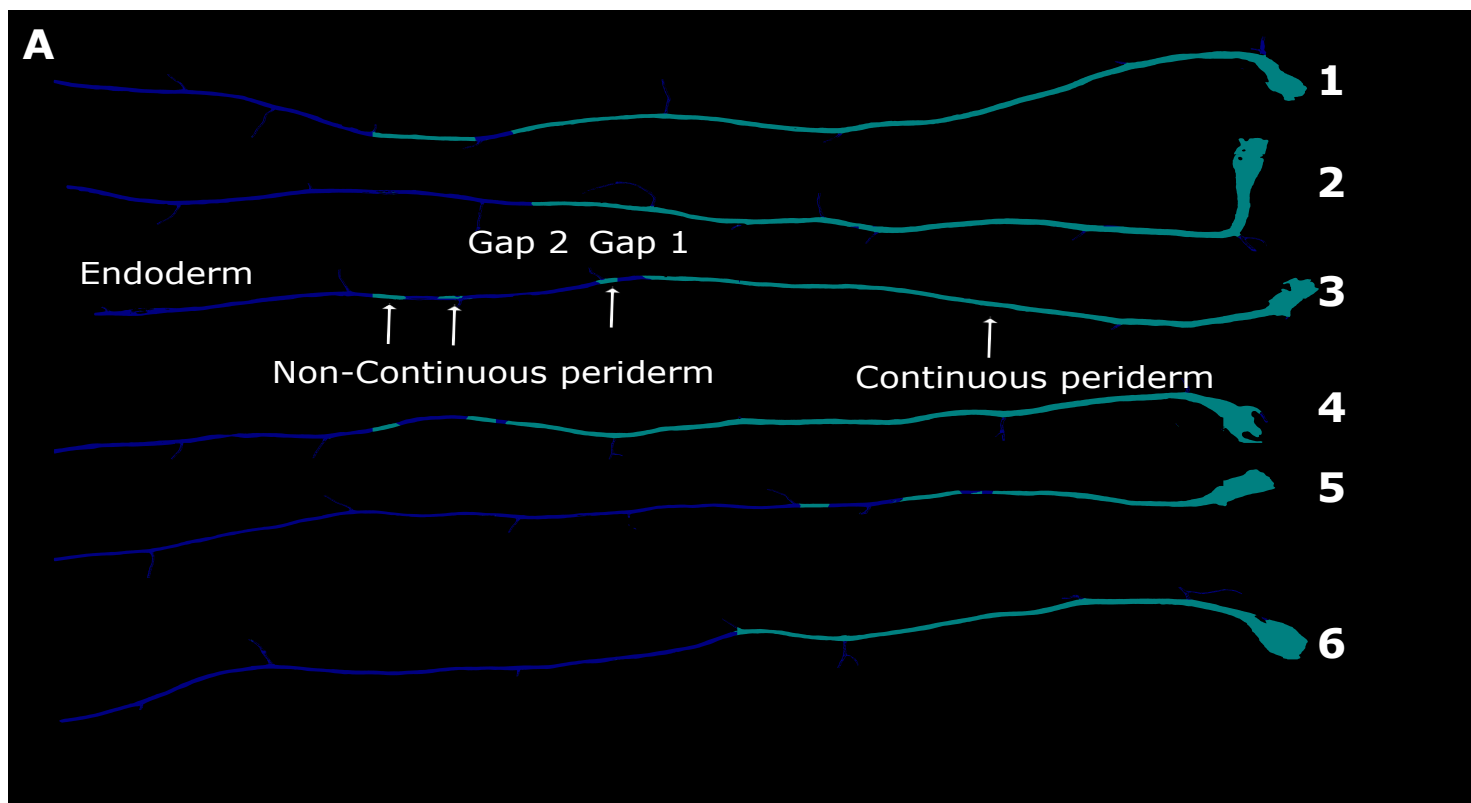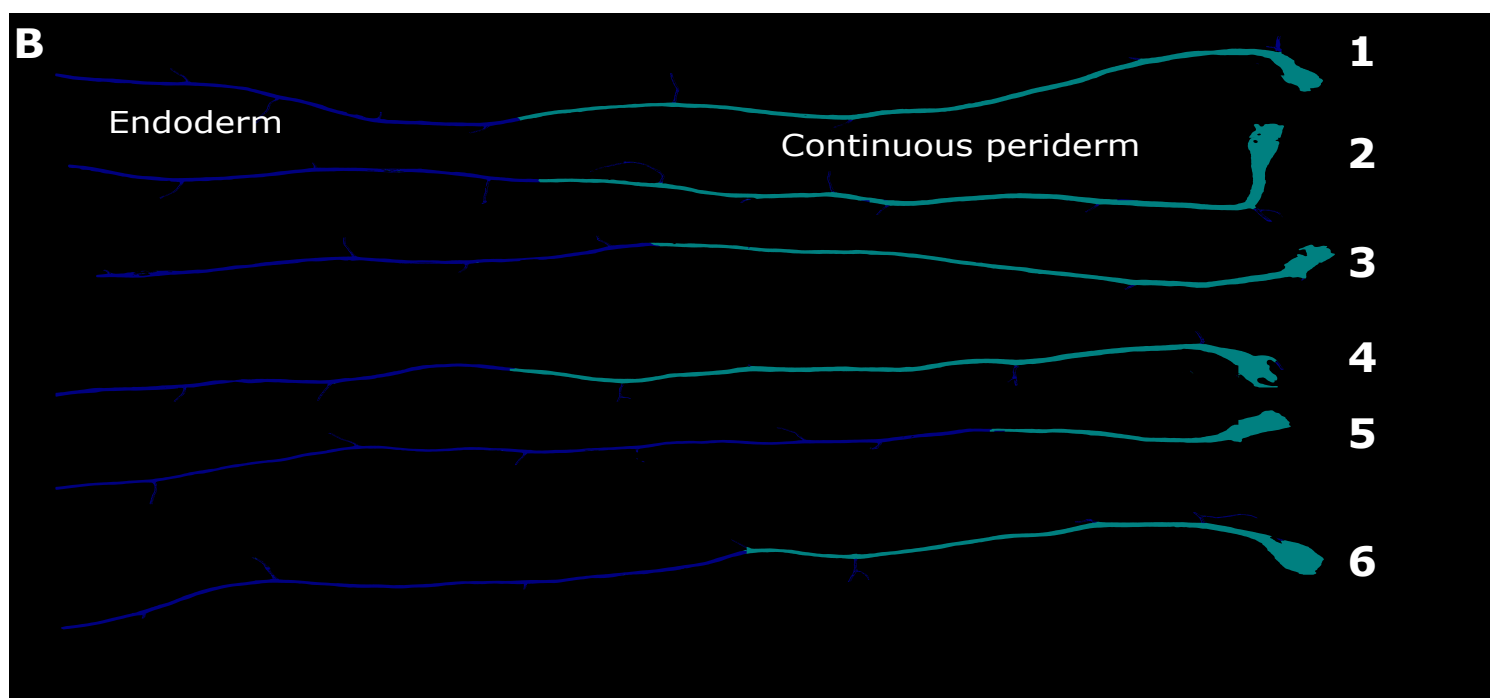

Supplement: Supplementary 1 — Figs. S1 to S7 Tables S1 to S4 [file plantphenomics.0156.f1.zip › Fig.S1.pdf]

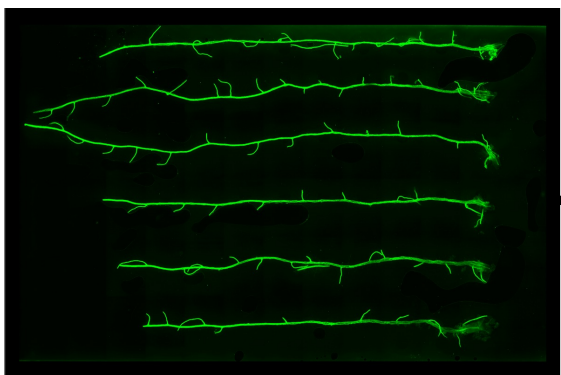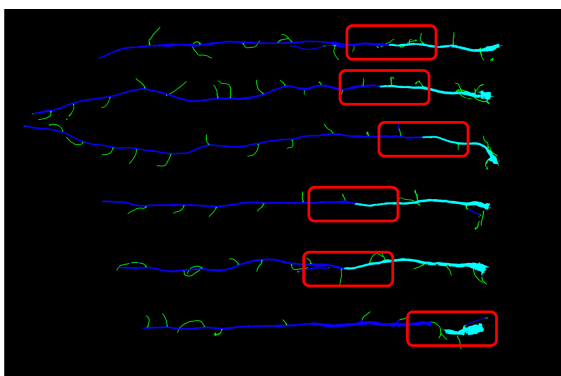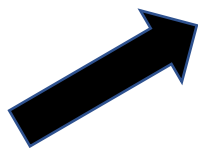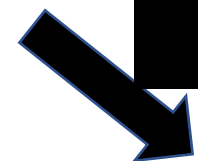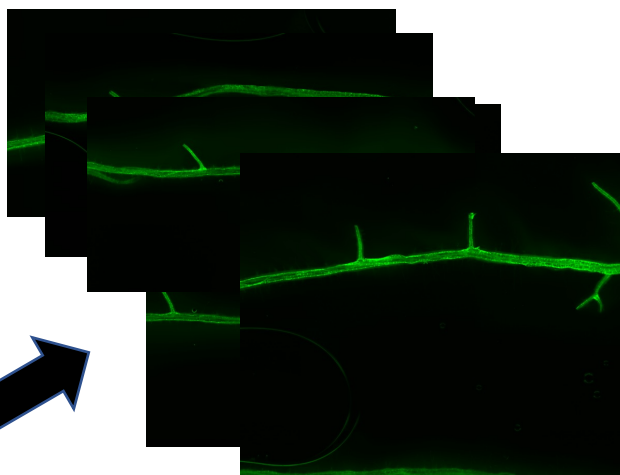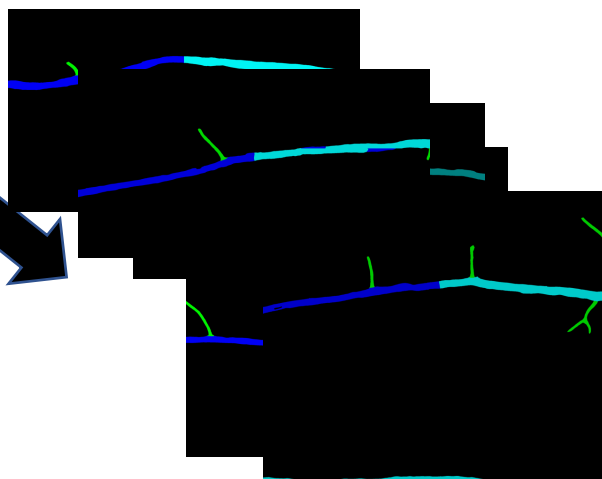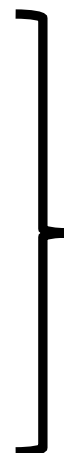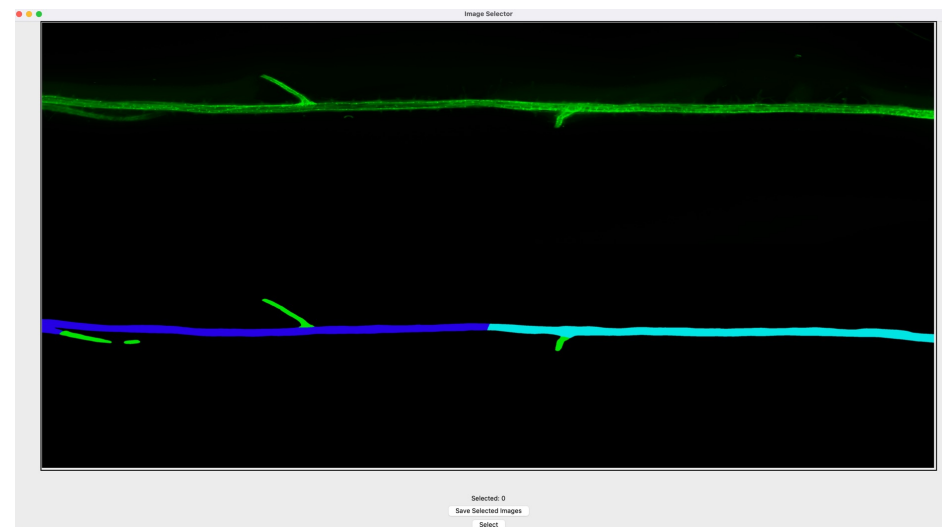

Supplement: Supplementary 1 — Figs. S1 to S7 Tables S1 to S4 [file plantphenomics.0156.f1.zip › Fig.S2.pdf]

**5\_24:**

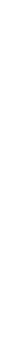

**23\_12:**

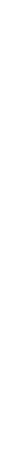

**18\_8:**

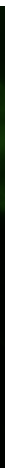

Supplement: Supplementary 1 — Figs. S1 to S7 Tables S1 to S4 [file plantphenomics.0156.f1.zip › Fig.S3.pdf]

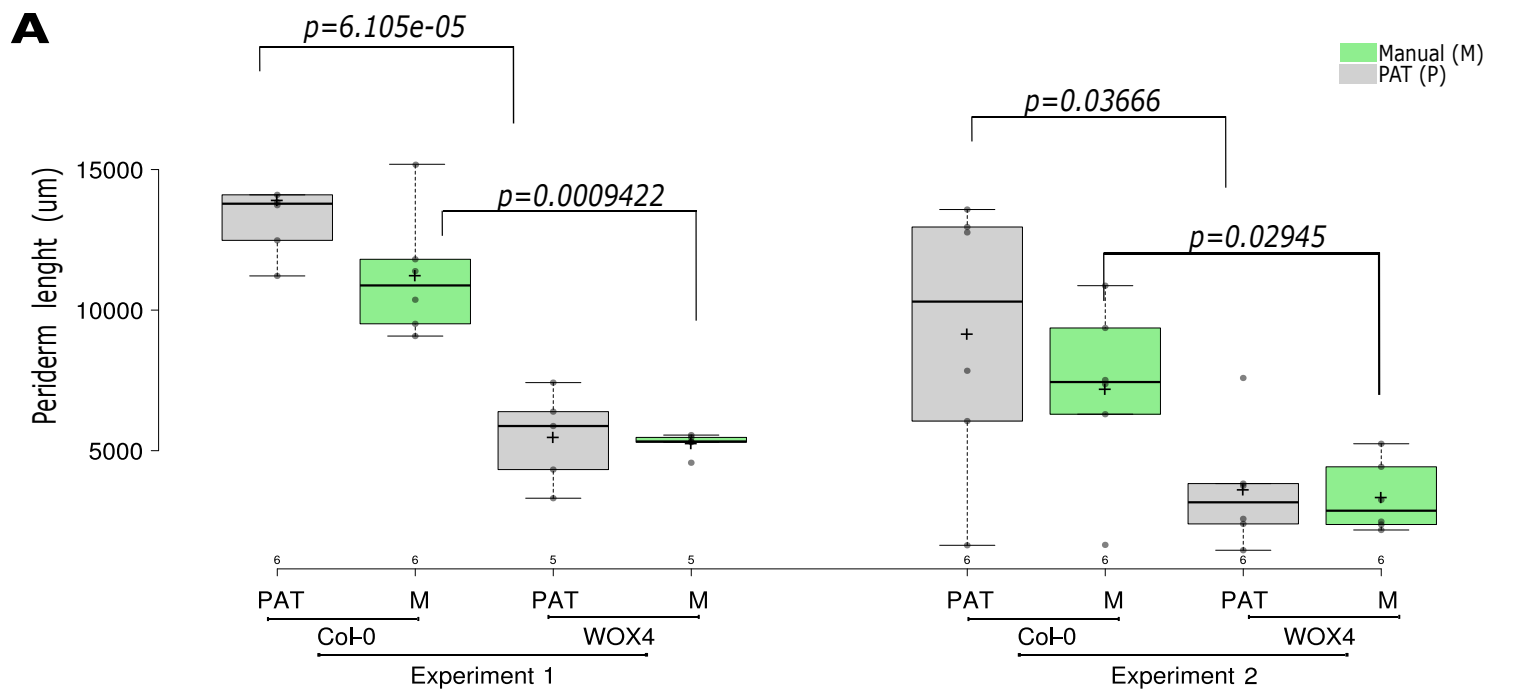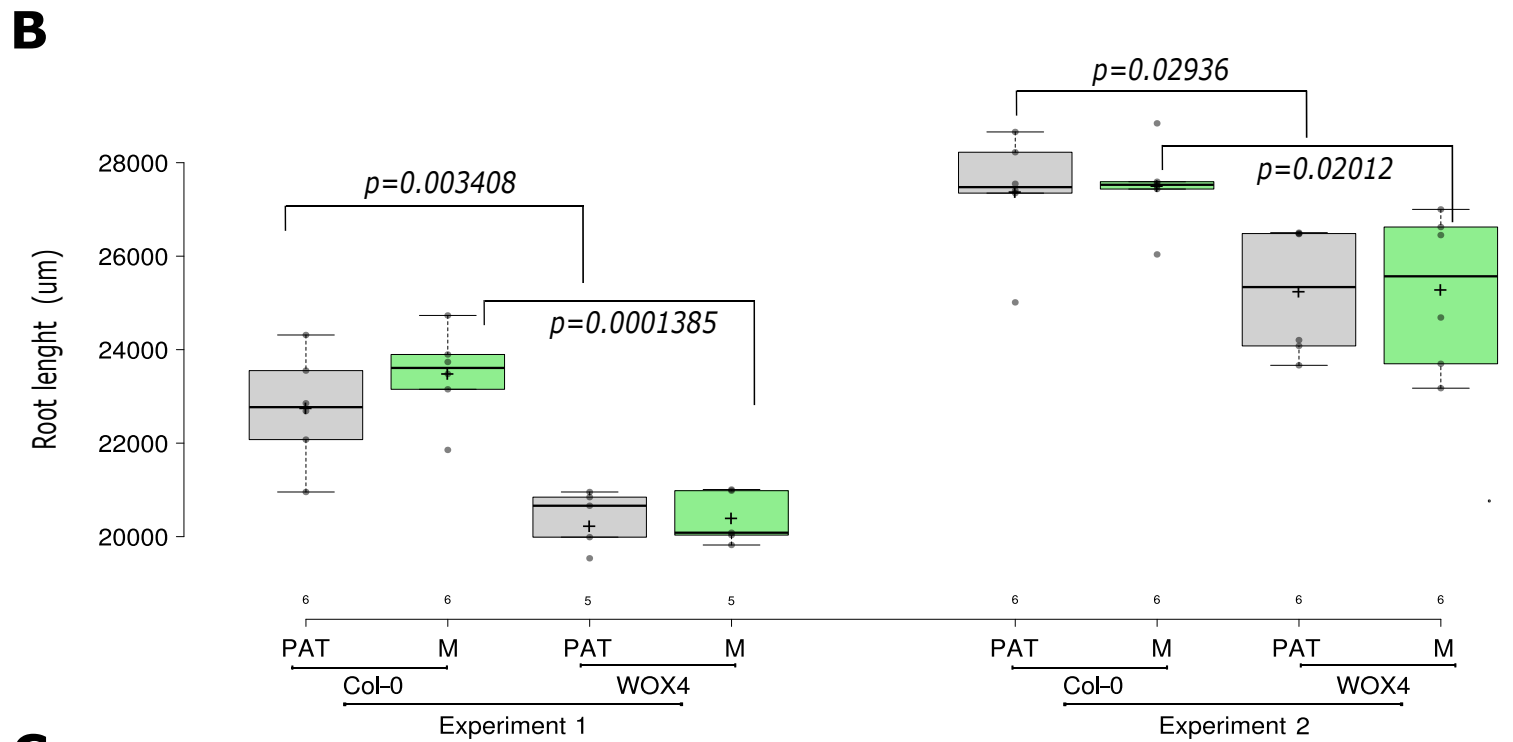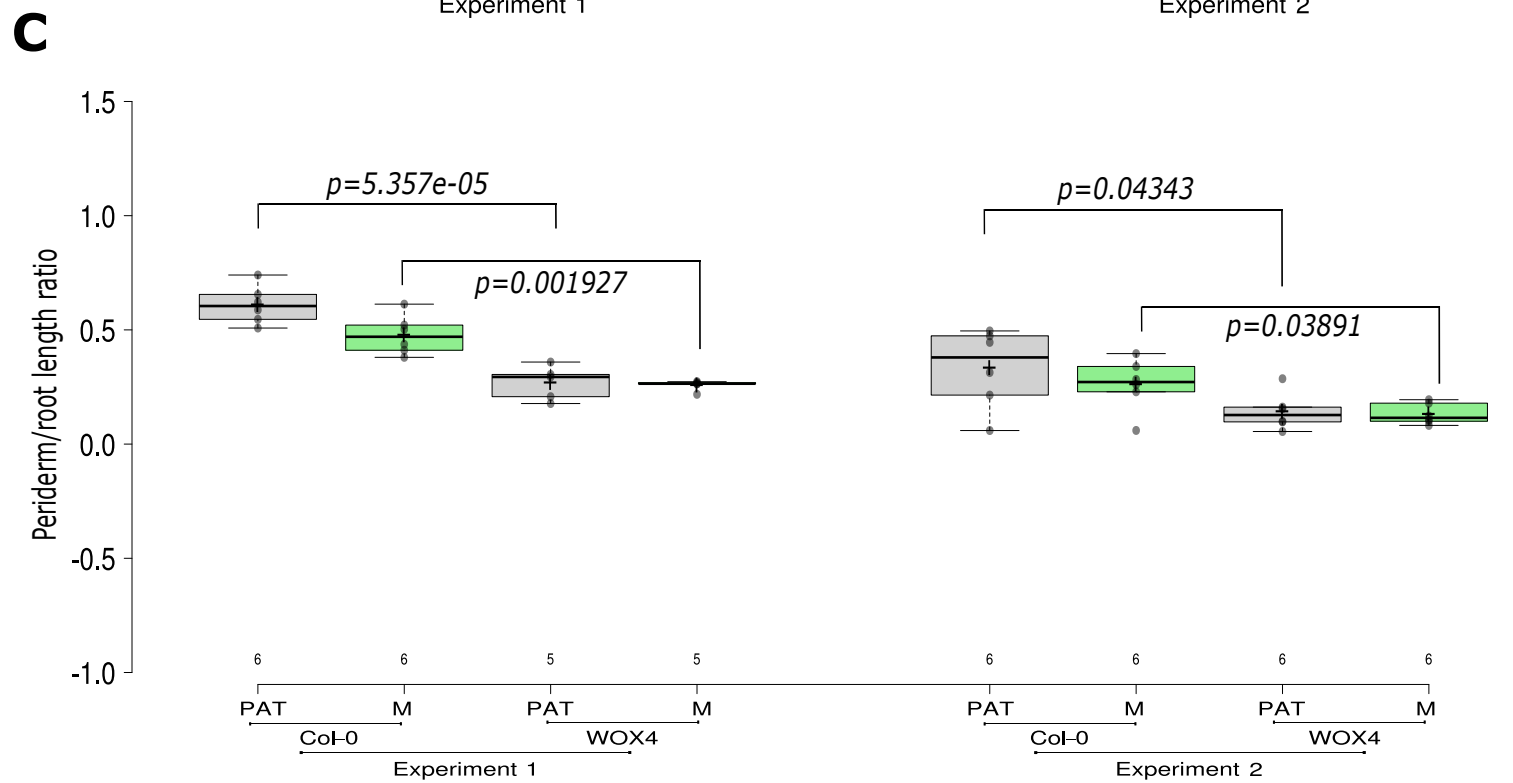

Supplement: Supplementary 1 — Figs. S1 to S7 Tables S1 to S4 [file plantphenomics.0156.f1.zip › Fig.S5.pdf]

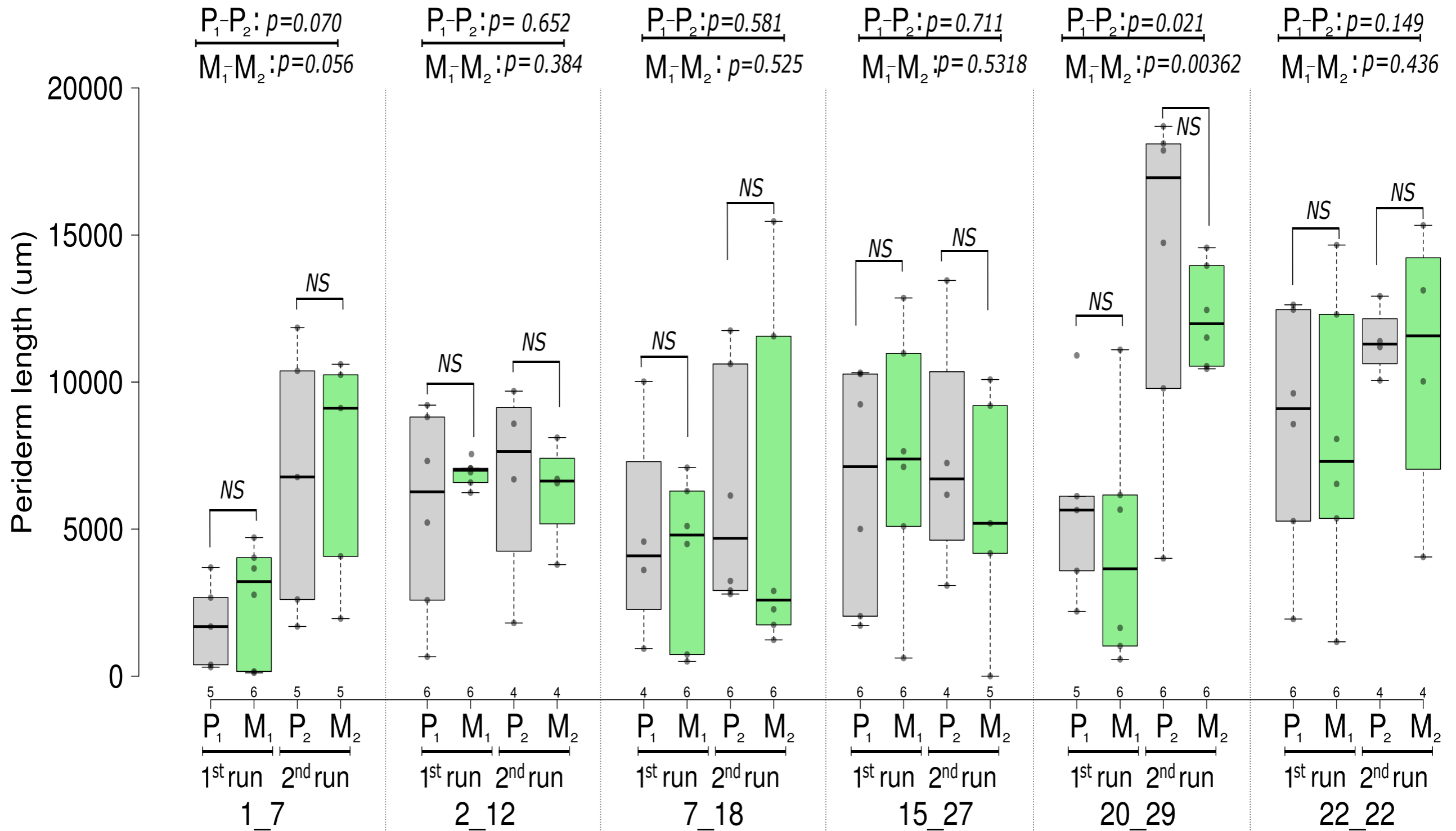





\*

$p=0.18$

$p=0.13$

$p=0.42$

A

$p=0.69$

$p=0.038$

B

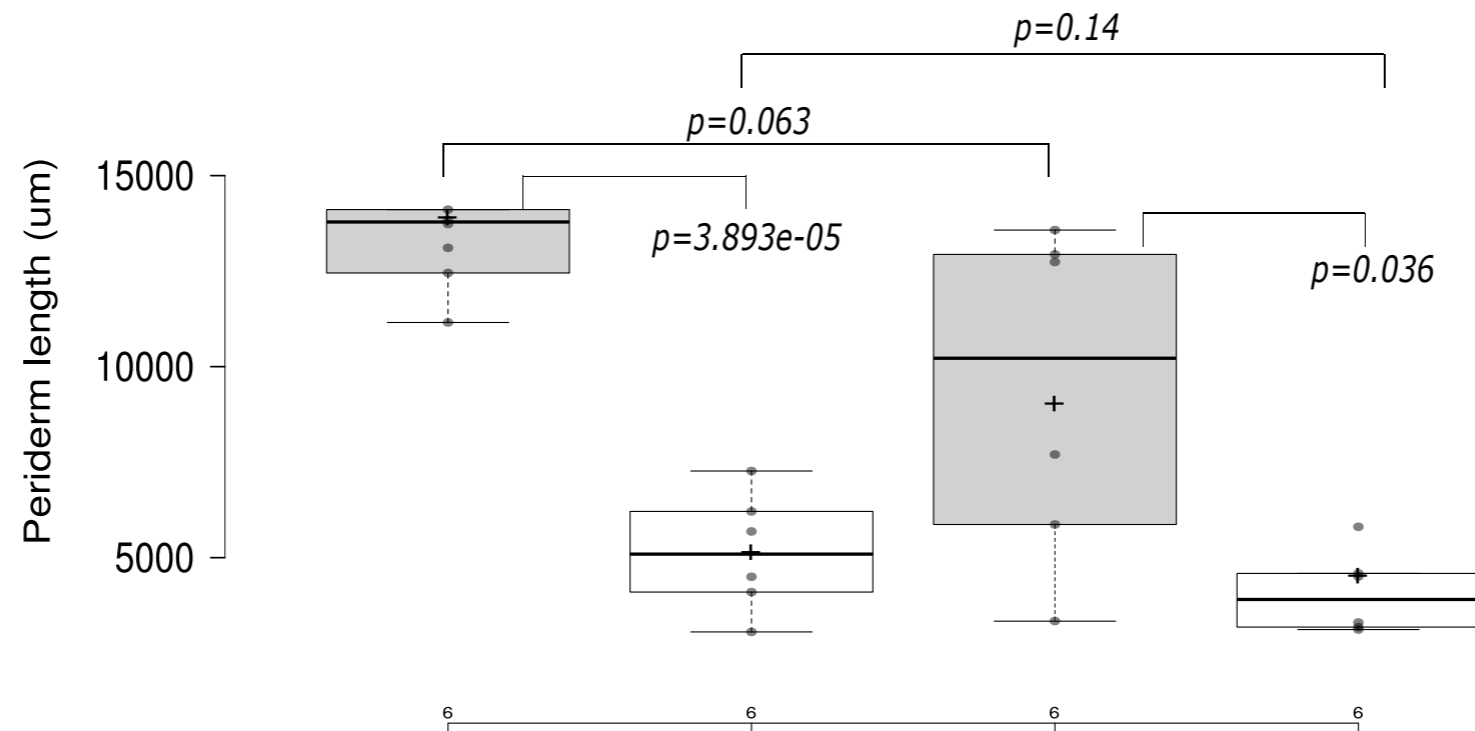

Supplement: Supplementary 1 — Figs. S1 to S7 Tables S1 to S4 [file plantphenomics.0156.f1.zip › Fig.S6.pdf]

A. PAT-1 vs Manual Measurement-1

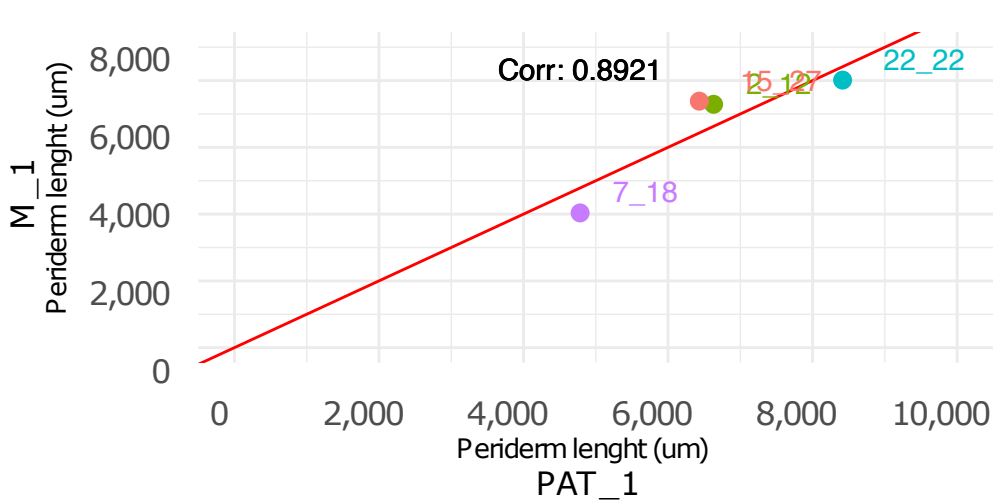

B. PAT-2 vs Manual Measurement-2

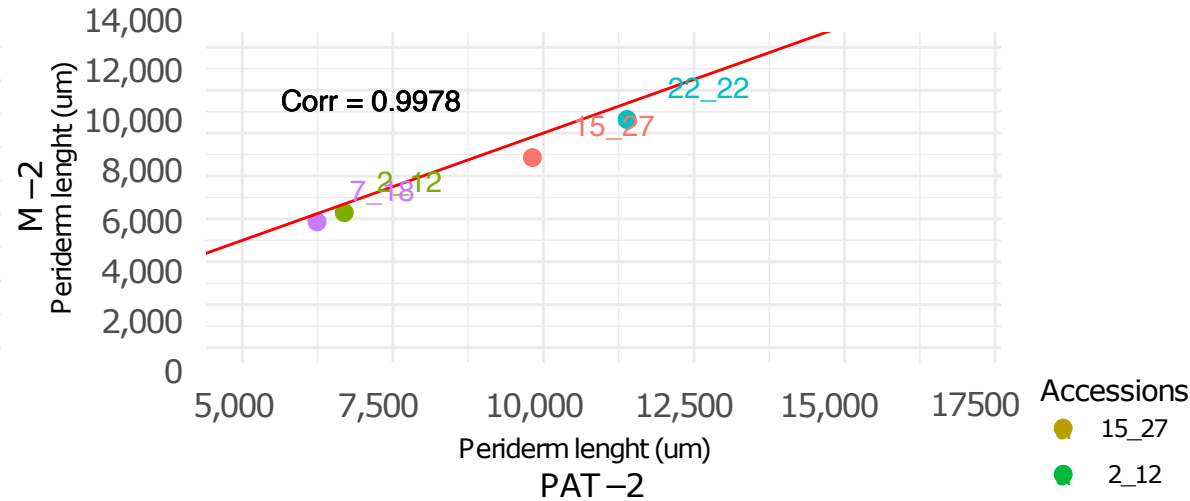

C. PAT-1 vs PAT-2

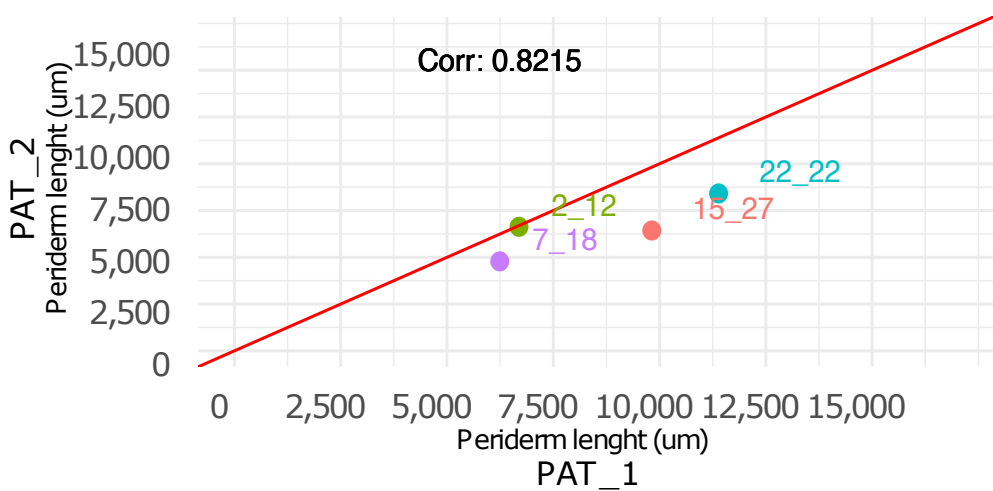

D. M-1 vs M-2

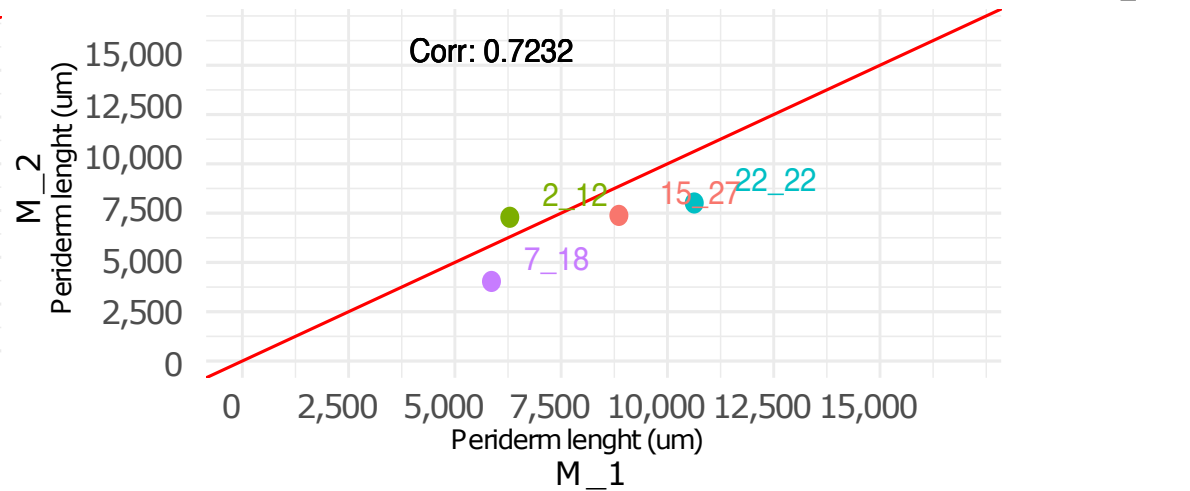

Supplement: Supplementary 1 — Figs. S1 to S7 Tables S1 to S4 [file plantphenomics.0156.f1.zip › Fig.S7.pdf]
